# Personalised Nutrition in Obesity and Prediabetes: Do Genotypes Matter?

**DOI:** 10.3390/nu18050815

**Published:** 2026-03-02

**Authors:** Magdalena Bossowska, Filip Bossowski, Edyta Adamska-Patruno, Katarzyna Maliszewska, Adam Krętowski

**Affiliations:** 1 Department of Endocrinology, Diabetology and Internal Medicine, Medical University of Bialystok, 15-276 Białystok, Poland; 2 Clinical Research Centre, Medical University of Bialystok, 15-276 Białystok, Poland

**Keywords:** nutrigenetics, obesity, prediabetes, diet

## Abstract

Background/Objectives: Obesity and prediabetes are overlapping global epidemics. This systematic review synthesises evidence on gene-diet interactions in adults with obesity, prediabetes, or related cardiometabolic risks. It evaluates Mediterranean and DASH dietary patterns, macronutrient quality, and energy restriction across both single-variant and polygenic score approaches. Methods: PubMed was searched for English language papers published in the last 5 years (last run: 31 October 2025). Fewer than 200 studies were retained after excluding those lacking explicit statistical testing for gene-diet interactions or relevant endpoints. Results: Evidence supports restricting saturated fat and preserving carbohydrate quality as general baseline targets, with associations heterogeneous by genotype. Effect modification was observed: healthy dietary patterns were associated with lower risk in high polygenic-risk strata (OR~0.53) but little or no benefit in low-risk groups. TCF7L2 variants were associated with macronutrient thresholds (e.g., protein > 18%, carbohydrate < 48%) affecting visceral adiposity, while APOA2 variants showed genotype-dependent inflammation, including paradoxical increases in markers with higher dietary antioxidant capacity. Interpretation was limited by underpowered interaction tests, multiplicity, and uneven ancestry representation (e.g., unique SLC16A11 and CREBRF signals). Conclusions: While anti-inflammatory dietary substitutions improve biomarkers irrespective of some variants (e.g., TCF7L2), genotype-informed nutrition appears to yield the largest absolute risk reduction in high-risk populations. Clinical implementation should therefore combine baseline diet-quality guidance with targeted strategies for genotype-specific response patterns (e.g., APOA2 antioxidant heterogeneity and TCF7L2 carbohydrate thresholds), rather than rely on uniform recommendations alone. Future progress requires preregistered, genotype-stratified trials and locally trained polygenic scores to address ancestry-specific genetic architecture.

## 1. Introduction

Obesity and prediabetes have caused overlapping global epidemics that impose major clinical and economic burdens. In Poland, a nationally representative 2019 survey using body mass index (BMI) values derived from self-reported height and weight measurements found that 59% of adults had excess body weight (overweight: 38%; obesity: 21%), whereas only 39% had normal weight, while the remaining 2% were classified as underweight [1]. Globally, approximately 18.5% of women and 14.0% of men have obesity [2]. Recent forecasting analyses suggest that, by 2050, nearly 60% of adults (about 3.8 billion people) will have overweight or obesity [3]. The prevalence of prediabetes varies by definition, with widely cited global figures near 10.5%; in 2024, the category-specific prevalence of impaired glucose tolerance was 12.0% (635 million adults, 20–79 years) and that of impaired fasting glucose levels was 9.2% (488 million adults). Direct health expenditures due to diabetes surpassed one trillion USD for the first time in 2024 (≈US $1.015 trillion; ~12% of global health expenditure) and are projected to continue to rise over the coming years, reaching ≈US $1.043 trillion by 2050 [4].

Nutrigenetics investigates whether genotype modifies the efficacy of specific dietary strategies, which may explain heterogeneous responses to equally “healthy” diets. These interactions may act through adiposity and energy balance, lipid profile, insulin–glucose homeostasis, and inflammation/redox biology in response to Mediterranean-style dietary patterns; carbohydrate quality (glycaemic index [GI], fibre content, and whole grains); fat quality (saturated fatty acids [SFAs], monounsaturated fatty acids (MUFAs), and polyunsaturated fatty acids [PUFAs]); protein intake; and energy restriction. The effects are expected to be modest and context-dependent across single-variant and polygenic score (PGS) frameworks [5,6,7].

Habitual diet differs substantially by region, shaping the baselines against which gene-diet effects are estimated. Mediterranean programmes operationalise a low SFA content (commonly ≤10% energy intake, sometimes approximately 8%), higher MUFA (approximately 21–22% energy) and PUFA (approximately 6–8% energy) contents, and fibre targets around approximately 26–30+ g/day [8,9,10,11,12]. Western and pan-European comparators often target total fat near approximately 30% energy with SFAs < 10% energy, and the World Health Organisation (WHO) guidance adds explicit limits on free sugars (<10% energy, with a conditional target < 5%) [13,14,15,16,17,18]. Across studies, adherence and exposure assessments have used the 14-item Prevención con Dieta Mediterránea (PREDIMED) score, food records, food frequency questionnaires (FFQs), repeated 24-h recalls, energy percentage thresholds and absolute cut-offs (e.g., SFA ≤ 23.2 g/day in South Asian cohorts), complicating direct comparisons and pooling [19,20]. Moreover, several methodological limitations remain unresolved: interaction tests are often underpowered [13,21,22]; multiplicity is inconsistently handled [10,23]; dietary exposures are variably defined and prone to measurement error; endpoint timing differs across studies; and representation across ancestries is uneven. These factors limit the transferability and calibration of PGSs across populations [24,25,26,27].

Contemporary definitions of prediabetes rely on fasting plasma glucose (FPG) levels, the results of the oral glucose tolerance test (OGTT), and, in some guidelines, glycated haemoglobin (HbA1c) levels. The Polish Diabetes Association define impaired fasting glucose as FPG levels of 100–125 mg/dL (5.6–6.9 mmol/L) and impaired glucose tolerance as 2-h OGTT values of 140–199 mg/dL (7.8–11.0 mmol/L), whereas the American Diabetes Association (ADA) additionally designates HbA1c levels of 5.7–6.4% as prediabetes [28,29]. These definitional discrepancies have critical implications for nutrigenetics; when entry criteria vary across cohorts, the same gene-diet interaction is effectively tested in populations with fundamentally different glycaemic baselines and trajectories.

The genetic architecture influencing dietary responses spans loci involved in multiple domains: (i) personalised eating behaviours and energy balance, including fat mass and obesity-associated protein (FTO) [30], melanocortin 4 receptor (MC4R) [31], and transmembrane protein 18 (TMEM18) [32]; (ii) β-cell function and incretin biology, including transcription factor 7-like 2 (TCF7L2) [24,33] and melatonin receptor 1B (MTNR1B) [34]; (iii) lipid handling, including apolipoprotein A2 (APOA2) [35], apolipoprotein A5 (APOA5) [9], lipoprotein lipase (LPL) [18], cholesteryl ester transfer protein (CETP) [18], fatty acid desaturase genes (FADS) [36], and apolipoprotein E (APOE) [37]; (iv) insulin resistance and adipogenesis, including peroxisome proliferator-activated receptor gamma (PPARG) [13]; and (v) ancestry-enriched signals such as solute carrier family 16 member 11 (SLC16A11) [15] and cAMP-responsive element binding protein 3 regulatory factor (CREBRF) [38]. Within this architecture, commonly tested dietary levers map onto relevant outcomes: variation in fat quality and carbohydrate quality aligns with changes in BMI and waist circumference (WC), as well as triglyceride (TG); high-density lipoprotein cholesterol (HDL-C); and low-density lipoprotein cholesterol (LDL-C) levels and glycaemic endpoints such as fasting glucose levels, homeostatic model assessment for insulin resistance (HOMA-IR) scores, HbA1c levels, and the OGTT results, with locus-specific contingencies and ancestry constraints [26,39,40,41].

**Figure 1:**
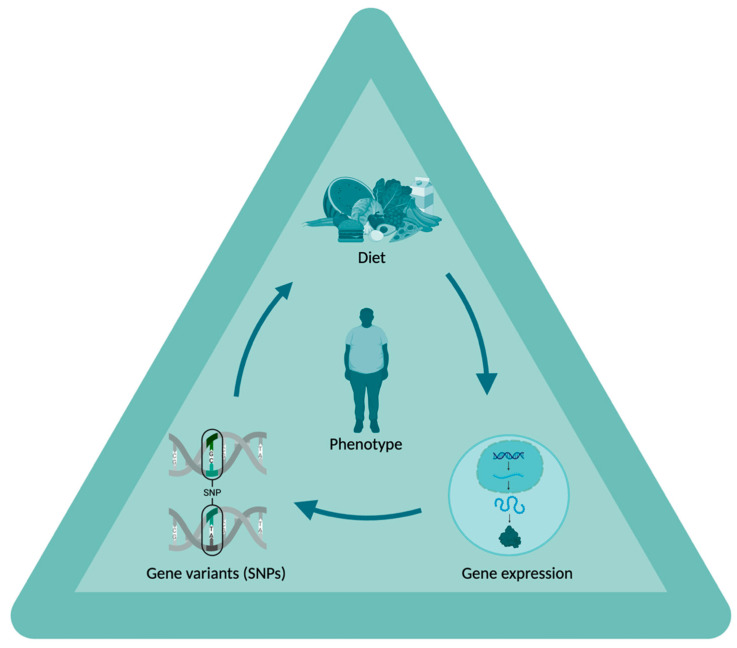
Diet–Genotype–Gene Expression Axis. This schematic illustrates the interactive relationships among dietary factors, genetic variation (single-nucleotide polymorphisms, SNPs), gene expression, and phenotype in a precision nutrigenetics framework. The phenotype is shown as the integrated outcome of these interacting components. Curved arrows indicate dynamic interrelationships and feedback among the components, rather than a linear sequence. Created in BioRender (accessed on 24 February 2026; web-based platform with continuous updates, version not specified). Available online: https://BioRender.com/gcpg338 (accessed on 24 February 2026).

Accordingly, this review evaluated the findings of human gene-diet interaction studies in adults with obesity, prediabetes, or related cardiometabolic risk, focusing on Mediterranean and Dietary Approaches to Stop Hypertension (DASH) patterns, carbohydrates, proteins, fat quality, and energy restriction. It considered single-variant and PGS approaches and appraised consistency, replication, and ancestry transferability while attending to exposure measurement and adherence. A schematic overview of the diet–genotype–gene expression axis underpinning this framework is shown in Figure 1.

## 2. Materials and Methods

**Figure 2:**
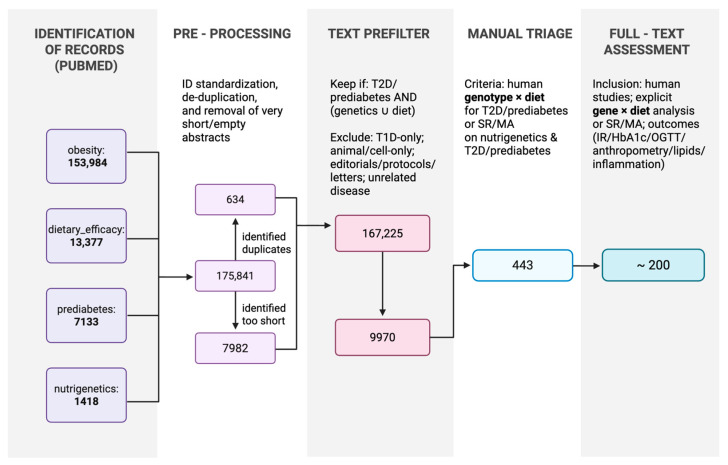
Identification, screening, eligibility, and inclusion (PRISMA-style flow). Records identified from PubMed in the last 5 years across four query families (total *n* = 175,912). Pre-processing included ID standardisation, de-duplication, and removal of very short/empty abstracts (see box for counts dropped). A lenient title/abstract prefilter requiring T2D/prediabetes AND (genetics OR diet) reduced the corpus to *n* = 9970 records. Manual title/abstract screening yielded *n* = 443 records for full-text review. Full-text assessment (English only) resulted in <200 studies meeting inclusion for synthesis. Created in BioRender (accessed on 24 February 2026; web-based platform with continuous up-dates, version not specified). https://BioRender.com/s8xgvdz (accessed on 24 February 2026).

We searched PubMed for studies conducted over a 5-year period (last run: 31 October 2025) using four query families: obesity (153,984 records), dietary efficacy (13,377), prediabetes (7133), and nutrigenetics (1418). The search yielded 175,912 records. After ID standardisation, we removed duplicates and concise or empty abstracts. A lenient, rule-based prefilter (case-insensitive regular expression over title and abstract) retained records mentioning T2D or prediabetes and at least one genetic or dietary signal, excluding type 1-only and animal/cell-only studies, editorials/protocols/letters, and studies on unrelated diseases; 9970 records remained. Manual title/abstract screening identified 443 records for full-text assessment. Full-text assessment was limited to articles in English, and fewer than 200 studies were retained after excluding those lacking explicit statistical testing for gene-diet interactions or relevant cardiometabolic endpoints. Eligible studies enrolled humans and addressed explicit genotype × diet interactions (or systematic reviews/meta-analyses on nutrigenetics with T2D/prediabetes) with outcomes including anthropometric measurements; glycaemic indices (fasting glucose/insulin levels, HbA1c levels, OGTT-derived measures such as corrected insulin response at 30 min [CIR30], and HOMA-IR scores); lipid levels; and inflammatory/oxidative stress markers. The full identification-to-inclusion workflow is presented in Figure 2.

## 3. Results

### 3.1. Genotype–Diet Effects on Body Composition

#### 3.1.1. Key Genes and Diet Exposures

Gene–diet interactions with body composition outcomes focus on a limited set of dietary factors that relate to loci implicated in energy balance and adiposity. These include fat quality [5,18,26], carbohydrate proportion and complexity [24,42], protein intake [31,43,44], Mediterranean-style dietary patterning [9,10], and energy restriction [45,46]. Polymorphisms in FTO and TCF7L2, alongside lipid-related genes such as APOA2 and apolipoprotein B (APOB), with additional signals at IRS1, are among the most frequently investigated factors in nutrigenetics. Pattern-level moderation by Mediterranean and DASH adherence also remains a central theme [9,24,40,45,47,48,49,50].

#### 3.1.2. TCF7L2 and Macronutrient Thresholds

TCF7L2 has been repeatedly examined as a moderator of diet and lifestyle effects across intervention and observational studies (Table 1). In a pooled analysis of seven RCTs (*n* = 4114), Huang et al. reported that diet/lifestyle interventions were associated with greater reductions in fasting glucose per copy of the TCF7L2 rs7903146 T risk allele (−3.06 mg/dL; 95% CI: −5.77 to −0.36; *p* = 0.028), while no significant interactions were observed for changes in body weight or waist circumference [45]. However, cross-sectional analysis found marginal significance for the rs7903146 association with waist circumference (TT: 83.5 ± 20.1 cm vs. CC: 80 ± 14.2 cm; *p* = 0.05) [50]. A broader synthesis by Hosseinpour-Niazi et al. provided context for these mixed findings: weight loss dietary RCTs with a more than one-year duration showed that serum glucose and insulin levels decreased and insulin resistance improved preferentially in non-risk allele subjects with overweight/obesity, whereas short-term RCTs (<10 weeks) generally showed no modification of glycaemic parameters by TCF7L2 genotype [40]. In cross-sectional analyses, macronutrient composition was a key determinant of visceral adiposity (Table 1). In a Polish cohort (*n* = 810), genotype-dependent thresholds were reported for visceral adiposity: TT carriers with higher protein intake (>18% energy) exhibited higher HbA1c (*p* = 0.038) and higher VAT/SAT ratios, in contrast to CC carriers, in whom obtaining ≥18% energy from protein was associated with lower visceral adipose tissue accrual. Conversely, CC carriers showed susceptibility to lower carbohydrate intake (≤48% energy), which was associated with higher VAT and lower SAT (*p* = 0.033) [24,45]. Finally, high fat intake (>30% energy) was associated with increased VAT in both CC (*p* = 0.012) and TT (*p* = 0.0006) genotypes [24].

#### 3.1.3. MC4R: Protein Sensitivity and Metformin Response

Cross-sectional evidence indicates genotype-contingent associations between macronutrient distribution—particularly the percentage of energy from protein—and central adiposity across MC4R variants (Table 2). In a Polish cross-sectional analysis (*n* = 810), carriers of the rs17782313 CC and rs12970134 AA risk genotypes deriving >18% of total energy from protein showed higher BMI, total body fat content, visceral adipose tissue (VAT), and VAT/subcutaneous adipose tissue (SAT) ratio. Regression modelling was concordant, with higher protein energy linked to higher BMI (Est. 5.74, R^2^ = 0.12), body fat content (Est. 8.44, R^2^ = 0.82), and VAT (Est. 32.59, R^2^ = 0.06) in these risk groups. In contrast, in protective genotypes (rs633265 GG and rs1350341 GG), more moderate protein intakes were reported alongside more favourable metabolic profiles, and diets providing >48% of energy from carbohydrates with <30% from fat were associated with lower body weight, waist circumference, and insulin resistance markers [31]. Beyond observational associations, pharmacologic co-intervention may further condition response (Table 2). In a Russian intervention study (Valeeva et al.), MC4R TT homozygotes receiving metformin plus diet therapy achieved significantly greater weight loss (−5.35 ± 0.89% vs. −2.5 ± 0.86%; *p* = 0.037) and fat mass reduction (−1.6 ± 0.28% vs. −0.65 ± 0.26%; *p* = 0.027) compared to CC/CT genotypes [13]. These findings suggest that, for MC4R genotypes, dietary patterning and pharmacological augmentation may jointly influence weight and fat mass outcomes.

#### 3.1.4. FTO: Fat Intake and Weight Loss Response

Evidence across observational and intervention studies suggests that FTO rs9939609 effects on adiposity and metabolic outcomes are context-dependent, with diet quality, intervention design, sex, and outcome choice affecting whether associations are detected (Table 3). In population-based studies, higher total fat intake has been reported to strengthen the association between the rs9939609 A risk allele and higher BMI [41,52,53]. Over 12 months of lifestyle or calorie restriction programmes, several reports have described smaller weight losses among A allele carriers than among TT carriers, although results varied substantially across cohorts [19,54]. These differences were reported to be attenuated with improved overall diet quality, including Mediterranean-style or higher protein/fibre eating patterns [42]. Additional data indicate heterogeneity by sex and anthropometric definition: in a Norwegian cohort with severe obesity (*n* = 97), the rs9939609 A allele was associated with lower total insulin sensitivity in males but not females [49]. Furthermore, a Mexican cross-sectional study (*n* = 684) found no significant genotype effect on BMI or body fat, yet TT carriers exhibited higher waist-to-height ratios (0.52 ± 0.07 vs. 0.49 ± 0.08), suggesting that FTO-associated signals may be more apparent in measures of central adiposity and specific metabolic endpoints than in BMI alone [47].

#### 3.1.5. PPARG: PUFA vs. SFA Effects

Evidence from intervention and synthesis studies indicates that PPARG variation can condition both the magnitude of weight loss and the pattern of central fat change during diet therapy, alongside fatty acid-specific gene-diet interactions (Table 4). In overweight/obese women with prediabetes, PPARG rs1801282 CC homozygotes achieved significantly greater weight loss with diet therapy alone (−2.92 ± 0.57% vs. −0.33 ± 0.70% in CG/GG carriers; *p* = 0.013) and more favourable changes in waist/hip ratio (−2.78 ± 0.97% vs. +0.70 ± 1.52%; *p* = 0.05), consistent with genotype-dependent differences in central adiposity response to standard dietary intervention [13]. Complementing these clinical findings, systematic review evidence suggests that the PPARG Pro12Ala polymorphism interacts with dietary fat type, with 12Ala carriers showing lower BMI on high polyunsaturated fatty acid (PUFA) diets but higher BMI on high saturated fatty acid (SFA) diets, suggesting that fat quality may modify PPARG-linked adiposity phenotypes [5].

#### 3.1.6. Polygenic and Genetic Risk Scores

Several studies used genetic risk scores (GRSs) or polygenic scores (PGSs) to capture aggregate susceptibility and assess gene-diet interactions across macronutrient exposures and dietary patterns (Table 5). In a Sri Lankan cohort (*n* = 105), a 10-SNP metabolic GRS interacted with polyunsaturated fatty acid (PUFA) intake on waist circumference (P-interaction = 0.00009). Among participants with high genetic risk (≥6 risk alleles), higher PUFA intake (≥3.1 g/day) was associated with lower waist circumference (*p* = 0.047) [55]. Similarly, in Asian-Indian participants carrying ≥2 CETP/LPL risk alleles, low saturated fat intake (≤23.2 g/day) was associated with smaller waist circumference (Beta = −0.01 cm; *p* = 0.03), whereas high saturated fat intake increased waist circumference in this genetic risk group (Beta = 0.02 cm; *p* = 0.02) [18]. Beyond fat quality, carbohydrate-related polygenic signals have also been reported: a vitamin D-related GRS interacted with carbohydrate quantity to influence body fat percentage, indicating that aggregate genetic susceptibility may modify adiposity responses to carbohydrate load [56]. Randomised comparisons stratified by a B12 GRS further suggested that protein effects can emerge at the aggregate-risk level: in the lower-risk strata, assignment to a high-protein diet was associated with lower waist circumference than assignment to a low-protein diet, with adherence to the assigned plan implicated as an important contributor to observed effects [42]. In the POUNDS Lost trial (*n* = 583), genetically determined adiposity subtypes (WHRonly+ PGS) significantly interacted with dietary protein on glycaemic traits, including fasting glucose (P-interaction = 0.0007) [43].

Characteristics of the included studies evaluating gene-diet interactions on adiposity are detailed in Table S1 [5,9,10,11,13,15,18,24,26,31,33,39,42,43,44,45,46,47,48,49,50,51,54,55,56,57,58,59,60,61,62,63,64,65,66,67,68,69,70,71,72,73,74,75,76,77,78,79,80,81,82,83,84,85].

**Table 5:** Evidence map of genetic and polygenic score × diet interactions for central adiposity and glycaemic traits. The table summarises each study by score composition, dietary exposure definition/threshold, outcome(s), and the direction of the score × diet interaction; effect estimates and interaction *p*-values are reported when available (GRS, genetic risk score; PGS, polygenic score; PRS, polygenic risk score; NR, not reported; Pint, interaction *p*-value).

Study (Year)	Score Composition	Exposure (Definition/Threshold)	Outcome(s)	Key Interaction Finding (Direction)	Effect Size/Estimate (If Reported)	*p*-Value
Sekar et al., 2025 [55]	10-SNP metabolic GRS	PUFA intake (≥3.1 g/day); high GRS (≥6 risk alleles)	WC	Lower WC in high GRS + high PUFA group	NR (WC lower; *p* = 0.047 noted in source text)	*P*_int_: 0.00009
Wuni et al., 2022 [18]	CETP/LPL 3-SNP GRS	SFA intake	WC	Low SFA reduced WC in high GRS	NR	*P*_int_: 0.006
Alathari et al., 2022 [56]	Vitamin D 8-SNP GRS	Fiber intake (low fibre stratum)	BMI	Higher BMI in high GRS + low fibre	NR	*P*_int_: 0.020
Alathari et al., 2022 [56]	Vitamin D 8-SNP GRS	Fat intake (low fat stratum)	HbA1c	Lower HbA1c in high GRS + low fat	NR	*P*_int_: 0.029
Chen et al., 2021 [43]	159-SNP adiposity PGS (WHR only+)	Protein intake	Fasting glucose	WHR only+ PGS × protein interaction reported	NR	*P*_int_: 0.0007
Padilla-Martinez et al., 2022 [86]	68-SNP T2D PRS	Observational	Fat mass	PRS associated with Δ fat mass	NR	*P*(reported): 0.025
Sekar et al., 2024 [59]	23-SNP GRS	MUFA intake (low MUFA stratum)	HbA1c	Higher HbA1c in high GRS + low MUFA	NR	*P*_int_: 0.026

### 3.2. Lipid Profile and Fatty Acid Metabolism: Fat Quality × Genotype Interactions

**Figure 3:**
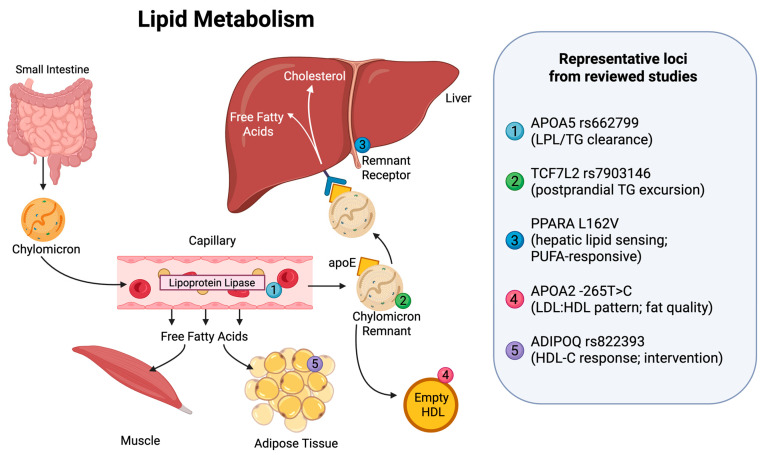
Conceptual mapping of genotype-dependent lipid responses in exogenous lipid transport and clearance. This schematic maps loci discussed in Section 3.2 to key steps in postprandial lipid handling, including chylomicron lipolysis, remnant formation and clearance, hepatic lipid processing, and HDL-related remodelling. Numbered labels denote example loci associated with genotype-dependent lipid responses in the reviewed studies (APOA5 rs662799, TCF7L2 rs7903146, PPARA L162V, APOA2 -265T>C, and ADIPOQ rs822393). Locus placement is conceptual and reflects the pathway step most relevant to the mechanistic or phenotypic association described in the review; it does not imply exclusive tissue-specific expression or localisation. The figure provides a conceptual synthesis of the reviewed evidence and is not an exhaustive pathway map. Created in BioRender (accessed on 24 February 2026; web-based platform with continuous up-dates, version not specified). https://BioRender.com/farppk6 (accessed on 24 February 2026).

Beyond generalised adiposity, genetic determinants of lipid profiles show locus-specific effects on triglyceride clearance and HDL remodelling [9,10,48,64]. Across diverse cohorts, fat quality (SFA vs. MUFA vs. PUFA) is associated with differences in these phenotypes that are not fully captured by weight loss alone [11,26,55,84]. Conceptually, the evidence in this section converges on a “fat load handling” framework in which lipid sensing (via nuclear receptors), lipid transport (via apolipoproteins), and postprandial clearance dynamics jointly shape genotype-contingent lipid responses (Figure 3).

#### 3.2.1. The Gatekeepers: Apolipoproteins and Nuclear Receptors

In Mediterranean cohorts, lipid-related loci, including apolipoprotein and adipokine genes, were associated with genotype-dependent differences in triglyceride and HDL-C responses, most clearly during MUFA-rich, hypocaloric interventions. At the ADIPOQ rs822393 locus, non-T-allele carriers (CC) achieved a greater HDL-C increase (Δ +8.9 ± 1.1 mg/dL) compared to T-allele carriers (Δ +1.7 ± 0.8 mg/dL; *p* = 0.02), while LDL-C reductions were comparable across genotypes (*p* = 0.41) [10]. Similarly, APOA5 rs662799 modified triglyceride responses: non-*C*-allele carriers showed a larger triglyceride decrease (Δ −19.3 ± 4.2 mg/dL) than C-allele carriers (Δ −3.2 ± 3.1 mg/dL; *p* = 0.02). This attenuated response in C-allele carriers has been interpreted as consistent with impaired lipoprotein lipase activity, which may limit triglyceride hydrolysis even under favourable dietary fat quality [9].

Mechanistic signals from nuclear receptor biology are consistent with this pattern of “gating” at the level of lipid sensing. PPARA L162V carriers exhibited PUFA-sensitive reductions in triglycerides and apolipoprotein C-III, consistent with PPAR-α-mediated VLDL clearance. Similarly, in an Iranian T2D cohort, APOA2 −265T>C homozygotes (CC) consuming higher SFA intakes displayed adverse LDL:HDL ratios that were attenuated when dietary fat quality improved [37].

#### 3.2.2. TCF7L2: Acute Fat Clearance and Lipemia

Beyond fasting levels, TCF7L2 has also been associated with postprandial fat handling. In Asian-Indian adults, rs7903146 T-allele carriers exhibited greater postprandial triglyceride excursions (higher 4h-TG and AUC; *p* < 0.01) following a standardised oral fat challenge [48]. This clearance profile may be diet-responsive: in a randomised comparison of Mediterranean and low-fat eating patterns, CC carriers demonstrated a coordinated circulating fatty-acid response, with significant genotype–diet interactions for Δ-SFA (*p* = 0.0046) and Δ-MUFA (*p* = 0.0078) that were not observed in the low-fat arm [11]. Collectively, these findings support genotype-contingent differences in postprandial lipaemia and fatty acid coordination within fat quality interventions, complementing the fasting lipid response signals described above [11,48].

#### 3.2.3. Metabolites and Fatty Acid Flux

Apart from traditional lipid panels, genotype-dependent variation extends to specific fatty acid metabolites. In large-scale analyses, the protective association of circulating *n*-3 PUFA was stronger in individuals carrying more DPA-associated alleles (P-interaction = 0.007) [26]. Furthermore, in a cohort enriched for the SLC16A11-risk haplotype, greater PUFA exposure was inversely associated with methylmalonylcarnitine levels (β: −0.038; *p* = 0.017), highlighting a metabolite axis relevant to this high-risk group [15]. Finally, epigenetic markers link total fat intake to CPT1A methylation (cg00574958), suggesting a potential mechanism connecting habitual fat exposure to triglyceride-related phenotypes [6].

Details of the included studies examining gene-diet interactions on lipid profiles and fatty acid metabolism are provided in Table 6 and Table S2 [9,10,11,13,14,15,18,19,21,24,26,31,33,34,45,46,47,48,49,50,51,55,56,57,58,59,64,65,70,71,72,75,78,84,87,88,89,90,91,92,93].

### 3.3. Insulin/Glucose Signalling: Risk Amplification and Pathway Specificity

Across loci implicated in insulin secretion and action, dietary quality acts as a modifier of genetic penetrance [25]. In general, lower saturated fat (SFA), higher-quality carbohydrates (e.g., whole grains and fibre), and higher unsaturated fat intake are associated with more favourable fasting glucose, HbA1c, and insulin sensitivity across many risk allele strata [43,51,84]. However, these associations are not uniform across genotypes. Available evidence supports a risk amplification pattern in which poorer dietary quality is associated with disproportionately adverse glycaemic phenotypes among individuals with the highest genetic susceptibility, whereas associations are attenuated or null in lower-risk strata [23,25].

#### 3.3.1. TCF7L2: Macronutrient Thresholds and Fat Sensitivity

TCF7L2 provides one of the most consistently replicated examples of macronutrient-dependent heterogeneity [51]. In rs7903146 T-allele carriers, higher SFA intake is associated with impaired insulin sensitivity [48,51], whereas replacing SFA with unsaturated fats—often within Mediterranean-style patterns—is associated with more favourable insulin sensitivity outcomes [11,51]. Macronutrient distribution also appears allele-specific within TCF7L2. At rs7901695, carbohydrate restriction to <48% of energy was associated with higher visceral adipose tissue (VAT) among CC carriers, whereas higher carbohydrate intake (>48% energy) was associated with lower HbA1c and a stronger early β-cell response (CIR30) among TT carriers [24]. These findings suggest that lower-carbohydrate approaches may not yield uniform benefits across TCF7L2 genotypes. In addition, sugar-sweetened beverage intake showed a graded interaction with rs7903146 on fasting glucose, with effects strongest in those with higher aggregate genetic risk [33].

#### 3.3.2. Polygenic Risk: The Amplification Effect

At the polygenic scale, aggregate genetic risk modifies the magnitude of diet-associated differences in glycaemic outcomes. In a 3-year study of prediabetic men, an unhealthy dietary pattern was associated with markedly higher T2D risk in the highest genetic risk stratum (OR 3.69), while a healthy dietary pattern in the same high-risk group was associated with risk reduction (OR 0.53); critically, dietary associations were null in the low-risk group [23]. Similarly, in a 5-year postpartum follow-up, a healthy lifestyle score reduced glycaemic abnormalities only among women in the highest polygenic risk tertile (OR 0.24), with null effects in lower-risk tertiles [57]. Collectively, these results suggest that precision nutrition strategies may yield the largest absolute risk reduction in genetically susceptible groups [23,57].

#### 3.3.3. Specific Macronutrient Tuning: Fat Quality and Carbohydrate Handling

Beyond aggregate risk, specific loci may modify metabolic responses to fat and carbohydrate quality. In SCD rs3071 CC carriers, replacing SFA-rich oils with MUFA-rich oils was associated with lower fasting glucose (+0.14 mmol/L with SFA vs. reduction with MUFA [84], supporting fat quality substitution as a modifiable dietary factor. In SLC16A11-risk haplotype carriers, higher PUFA exposure was inversely associated with methylmalonylcarnitine, a metabolite linked to adverse lipid metabolism. For carbohydrate handling, AMY1 copy number variation interacts with habitual starch intake to modify fasting glucose [78], while genome-wide interaction studies implicate loci near TRPM2/TRPM3 in HbA1c modification by carbohydrate-containing food groups [25].

#### 3.3.4. Pathway Specificity: Insulin Resistance vs. β-Cell Function

Evidence suggests that gene-diet interactions may preferentially affect specific physiological pathways. In the POUNDS Lost trial, an adiposity-linked polygenic score (WHR-PGS) interacted with dietary protein to modify β-cell compensation (HOMA-B), with less consistent modification of insulin resistance (HOMA-IR) [43]. Conversely, in the Diabetes Prevention Program, a partitioned polygenic score capturing β-cell burden predicted declining function independent of intensive lifestyle or metformin allocation [64]. Together, these findings suggest that insulin resistance phenotypes may be more responsive to dietary modification, whereas genetically mediated β-cell dysfunction may be less responsive to standard lifestyle interventions [64,94].

Comprehensive details of the included studies examining gene-diet interactions on insulin and glucose signalling are available in Table 7 and Table S3 [5,9,10,11,13,15,17,18,19,24,25,26,31,33,39,41,43,44,46,48,50,51,55,56,57,58,59,62,64,65,70,75,78,84,85,90,95,96,97,98,99,100,101].

### 3.4. Inflammation and Oxidative Stress: Redox Gating and Uncoupled Responses

Whereas adiposity and glycaemic control may show broad diet–genotype effect modification, diet–inflammation associations appear more locus-specific (Table 8), with variants in pathways implicated in redox balance and adipokine regulation influencing inflammatory responses [10,35,51,82,102,103]. Available evidence suggests that genetic variation may determine whether dietary exposures, such as antioxidant capacity [102,103] or dietary acid load [82], are associated with more pro-inflammatory versus anti-inflammatory profiles [82,102,103,104].

#### 3.4.1. The APOA2 Paradox: Redox Gating

A prominent example of genotype-dependent inflammatory response involves the APOA2 −265T>C (rs5082) locus, where genotype modifies both the direction and magnitude of biomarker associations with dietary exposures [35,102]. In patients with type 2 diabetes, APOA2 C-allele carriers consuming a diet with high renal acid load (PRAL) exhibited higher hs-CRP, leptin, and ghrelin levels, whereas no association was observed in T-allele carriers (P-interaction = 0.04) [35]. In contrast, the biomarker response to dietary total antioxidant capacity (DTAC) differed in the opposite direction by genotype: in T-allele carriers, higher DTAC was associated with lower hs-CRP and higher superoxide dismutase activity, whereas, in CC homozygotes, higher dietary antioxidant capacity was associated with higher interleukin-18 (IL-18) (*p* = 0.037) and lipid peroxidation markers (PGF2alpha). These findings suggest that, in APOA2 CC carriers, higher DTAC may not translate into the expected antioxidant biomarker profile, potentially reflecting genotype-dependent differences in redox or inflammatory regulation [102].

#### 3.4.2. Adipokines and the Mediterranean Effect

Genetic variation may modify adipokine responses to dietary intervention. At the ADIPOQ rs822393 locus, genotype modified responses to a hypocaloric Mediterranean intervention. Non-T-allele carriers (CC) showed an increase in adiponectin alongside improvements in HDL-C and insulin sensitivity, whereas T-allele carriers showed a smaller adiponectin response, suggesting limited adipokine responsiveness to dietary fat modification in this genotype group [10].

#### 3.4.3. Systemic Defence: Polygenic Antioxidant Response

At the polygenic level, genetic burden in antioxidant defence pathways may modify T2D risk. A polygenic risk score (PRS) constructed from antioxidant defence genes (e.g., GSTA5 and GPX1) showed a significant interaction with dietary exposures. Higher consumption of dietary antioxidants, vitamin C, and coffee was associated with attenuation of the T2D risk associated with a high genetic burden. Collectively, these findings suggest that exogenous antioxidant-related dietary exposures may partially offset higher inherited risk linked to reduced endogenous antioxidant capacity [103].

#### 3.4.4. TCF7L2: The “Override” Signal

Not all genetic risk signals extend to inflammatory outcomes [51]. While TCF7L2 rs7903146 modifies glycaemic responses, available evidence suggests no genotype-dependent differences in diet-related inflammatory biomarker changes in the setting examined [40]. In a randomised trial comparing a legume-based DASH diet with a standard DASH diet, the legume intervention reduced the hs-CRP, TNF-α, and IL-6 levels, and this anti-inflammatory effect was observed irrespective of TCF7L2 genotype [105]. These findings suggest that, even when glycaemic responses vary by TCF7L2 genotype, inflammatory biomarkers may remain responsive to dietary substitution in this context.

## 4. Discussion

Across the included studies, several findings aligned in a practical direction, but effects were frequently modified by genotype and context. Restricting saturated fatty acids (SFAs) while emphasising monounsaturated/polyunsaturated fatty acids (MUFAs/PUFAs), together with preserving carbohydrate quality, was associated with more favourable glycaemic and inflammatory profiles. Clinically relevant effect modification was evident in two ways: first, at the polygenic level, dietary quality may differentiate risk most strongly in genetically susceptible strata; and second, at selected loci, macronutrient “threshold” patterns and paradoxical biomarker responses argue against uniform prescriptions. In high-risk polygenic strata, an unhealthy dietary pattern was associated with markedly higher T2D risk (OR 3.69), whereas a healthy dietary pattern in the same stratum was associated with a substantial risk reduction (OR 0.53); in contrast, dietary associations were often null in low-risk groups [18,24,26,50,55,106,107,108]. This pattern supports prioritising adherence and diet quality, particularly in individuals with higher inherited susceptibility, while interpreting null findings in low-risk strata with caution, given limited power for interaction testing.

Within this baseline, the most consistent findings suggest several locus-specific patterns that can inform practice. For TCF7L2, macronutrient allocation appeared to matter beyond generic diet labels: in rs7901695, carbohydrate allocation around approximately ≤48% versus >48% of energy differentiated visceral adiposity and related glycaemic phenotypes by genotype, and higher SFA exposure was associated with worse insulin sensitivity in rs7903146 T-allele carriers [24,48,68]. These findings argue against assuming that aggressive carbohydrate restriction is uniformly favourable across TCF7L2 backgrounds. Inflammatory and oxidative stress outcomes also did not align uniformly with “healthier” exposures across genotypes. At APOA2−265T>C, higher dietary total antioxidant capacity was associated with higher inflammatory and lipid peroxidation markers in CC homozygotes (including higher IL-18 and PGF2α), whereas the expected anti-inflammatory pattern was observed in T-allele carriers, consistent with genotype-dependent uncoupling between antioxidant exposure and biomarker response [102]. By contrast, some pragmatic substitutions appear to confer benefits across genotypes in specific settings: in a DASH context, a legume-based dietary substitution reduced hs-CRP, TNF-α, and IL-6 and improved glycaemic indices, with the anti-inflammatory signal observed irrespective of TCF7L2 genotype in the examined trial [40]. Overall, the evidence supports a pragmatic clinical interpretation: maintain broadly supported diet-quality targets (SFA restriction, MUFA/PUFA emphasis, and carbohydrate quality) and use genotype primarily to avoid biologically discordant extremes at loci where replicated signals suggest threshold-like effects or paradoxical biomarker responses, rather than to mandate highly individualised diets for all patients [24,40,48,102].

Ancestry and population context frame interpretation and transferability. Replication and ancestry coverage are uneven, and several signals—particularly from single cohorts—should be interpreted cautiously. Nonetheless, the synthesis highlights population-specific genetic architectures that may not generalise from predominantly European evidence. In Mexican ancestry cohorts, SLC16A11-related findings in this set centred on a lipotoxic/metabolomic profile (including methylmalonylcarnitine) rather than clinical endpoints, suggesting distinct mechanistic targets for dietary modulation within that genetic background [15]. In Pacific peoples, CREBRF has been associated with a “favourable adiposity” architecture in which higher BMI does not translate proportionally into diabetes risk, challenging weight-centric prevention targets and supporting a more phenotype-calibrated approach to cardiometabolic risk assessment in these populations [38]. In Asian and Arab cohorts, several nutrigenetic signals—including TCF7L2-related associations—are informative but often drawn from limited settings, reinforcing the need for locally trained polygenic scores (PGSs) and genotype-stratified trials across ancestries to improve calibration and equity of deployment [15,18,19,24,38,47,50,51,75,109].

Interpretation of the reviewed evidence is constrained by small-to-moderate sample sizes for interaction testing, reliance on FFQs and pattern scores for exposure assessment, heterogeneity in intervention duration and co-interventions, and inconsistent handling/reporting of multiplicity, all of which increase between-study variability. Several findings are ancestry- or disease status-specific, limiting external validity, and single-cohort signals should be treated as hypothesis-generating until replicated [18,19,24,48,64,109]. Clinically, a defensible starting point remains SFA restriction with an emphasis on MUFA/PUFA and preservation of carbohydrate quality; within this framework, cautious macronutrient tailoring may be considered at selected loci where threshold patterns or discordant biomarker responses are reported (e.g., avoiding aggressive carbohydrate restriction in TCF7L2 rs7901695 CC; recognising APOA2-related heterogeneity in antioxidant-linked inflammatory biomarkers) while prioritising realistic, sustainable food substitutions that improve adherence and deliver genotype-robust gains [24,26,52,85,105,109].

## 5. Conclusions

This systematic review supports a clinically usable synthesis of nutrigenetic evidence in adults with obesity, prediabetes, or related cardiometabolic risk. Across study designs, the most reproducible baseline targets remain the restriction of saturated fatty acids (SFAs), with emphasis on monounsaturated/polyunsaturated fatty acids (MUFAs/PUFAs) and preservation of carbohydrate quality. Within this framework, the evidence most consistently supports a “risk amplification” model: individuals with the highest polygenic burden are not “non-responders” to dietary change but are often the highest responders to dietary quality and adherence. These groups show disproportionately higher glycaemic risk with unhealthy patterns (OR 3.69) and meaningful risk reduction with healthy patterns (OR 0.53), whereas associations are frequently attenuated or null in lower-risk strata.

Beyond broad diet quality, replicated locus-specific patterns indicate that genotype is most useful for avoiding biologically discordant extremes rather than prescribing highly individualised diets. Aggressive carbohydrate restriction (<48% energy) appears unfavourable for TCF7L2 rs7901695 CC carriers, given associations with higher visceral adiposity and less favourable glycaemic phenotypes relative to higher carbohydrate allocation. Similarly, in inflammation/redox biology, APOA2−265T>C (rs5082) CC homozygosity is associated with an uncoupled biomarker profile where higher dietary total antioxidant capacity correlates with higher inflammatory and lipid peroxidation markers, arguing against assuming uniform anti-inflammatory benefit from antioxidant-heavy patterns in this subgroup.

Emerging signals in Mexican (SLC16A11 lipotoxic phenotype) and Pacific (CREBRF “favourable adiposity” architecture) cohorts further suggest that biological targets and transferability differ by ancestry. This supports the need for locally trained polygenic scores and preregistered, adequately powered genotype-stratified trials that test realistic food substitutions across diverse populations. Pending such evidence, a pragmatic clinical approach is to apply robust diet-quality guidance broadly, use genetic information to identify likely high responders, and avoid the best-supported genotype–diet mismatches.

## Figures and Tables

**Table 1:** Evidence map of TCF7L2 genotype–diet interactions for adiposity-related outcomes. The table summarises each study by variant, dietary exposure definition/threshold, adiposity outcome(s), and the direction of the genotype × exposure interaction; effect estimates and interaction *p*-values are reported when available (NR, not reported; NS, not significant; NA, not applicable).

Study (Year)	Variant	Exposure (Definition/Threshold)	Outcome(s)	Key Interaction Finding (Direction)	Effect Size/Estimate (If Reported)	*p*-Value
Huang et al. 2021 [45]	rs7903146	Diet/lifestyle intervention	Body weight	No significant genotype × intervention interaction for body weight	NR	NS (NR)
Huang et al. 2021 [45]	rs7903146	Diet/lifestyle intervention	Waist circumference (WC)	WC reduction reported regardless of genotype; no significant interaction	NR	NS (NR)
Bauer et al. 2021 [24]	rs7901695	High protein (>18% energy)	VAT; VAT/SAT ratio	TT carriers showed higher VAT with high protein	NR	*p* (reported): 0.038
Bauer et al. 2021 [24]	rs7901695	Lower carbohydrate (≤48% energy)	VAT; SAT	CC carriers showed higher VAT with low CHO	NR	*p* (reported): 0.033
Bauer et al. 2021 [24]	rs7901695	High fat (>30% energy)	VAT; VAT/SAT ratio	Both genotypes showed higher VAT with high fat	NR	*p* (reported): 0.0006
Al-Odinan et al. 2025 [50]	rs7903146	Total energy	WC	TT carriers had the highest WC	83.5 cm (TT)	*p* (reported): 0.05
Hosseinpour-Niazi et al. 2022 [51]	Multiple	Weight-loss diets (review)	Insulin resistance	Non-risk allele subjects improved more (direction heterogeneous across studies)	NR	NA (review; varies)

**Table 2:** Evidence map of MC4R genotype–diet and diet–metformin interactions for adiposity and cardiometabolic outcomes. The table summarises each study by variant and dietary exposure definition/threshold, including metformin co-intervention where applicable, outcome(s), and the direction of the genotype × exposure interaction; effect estimates and interaction *p*-values are reported when available (NR, not reported; Pint, interaction *p*-value).

Study (Year)	Variant	Exposure (Definition/Threshold)	Outcome(s)	Key Interaction Finding (Direction)	Effect Size/Estimate (If Reported)	*p*-Value
Valeeva et al., 2022 [13]	rs17782313	Diet + metformin (vs. diet alone)	Weight loss	TT homozygotes showed greater weight loss	−5.35 ± 0.89% vs. −2.5 ± 0.86%	*p* (reported): 0.037
Valeeva et al., 2022 [13]	rs17782313	Diet + metformin (vs. diet alone)	Fat mass	TT homozygotes showed greater fat-mass reduction	−1.6 ± 0.28% vs. −0.65 ± 0.26%	*p* (reported): 0.027
Adamska-Patruno et al., 2021 [31]	rs17782313	High protein (>18% energy)	VAT; VAT/SAT ratio	CC carriers had higher VAT/VAT:SAR with higher protein strata	NR	*p* (reported): <0.05
Adamska-Patruno et al., 2021 [31]	rs12970134	High protein (>18% energy)	BMI; body fat	AA carriers had higher BMI and body fat with high protein	NR	*p* (reported): <0.05

**Table 3:** Evidence map of FTO rs9939609 interactions with diet/lifestyle factors for insulin sensitivity and anthropometric outcomes. The table summarises each study by exposure definition (e.g., meal test, dietary composition, physical activity, or Mediterranean diet adherence); outcome(s); and the direction of the genotype × exposure interaction; effect estimates and interaction *p*-values are reported when available (NR, not reported; NS, not significant; Pint, interaction *p*-value).

Study (Year)	Variant	Exposure (Definition/Threshold)	Outcome(s)	Key Interaction Finding (Direction)	Effect Size/Estimate (If Reported)	*p*-Value
De Soysa et al., 2021 [49]	rs9939609	Meal test	Total insulin sensitivity (IS)	A allele associated with lower total IS in males only; no effect in females	NR	NS (NR) for females; *p* NR for males
Sepulveda-Villegas et al., 2025 [47]	rs9939609	Diet composition	Waist-to-height ratio (WHtR); BMI	No genotype effect on BMI/body fat, but TT carriers had higher WHtR	WHtR: 0.52 ± 0.07 vs. 0.49 ± 0.08	NS (NR) for BMI
AlAnazi et al., 2024 [41]	rs9939609	Physical activity	BMI	Significant interaction reported	NR	P_int_: 0.02
AlAnazi et al., 2024 [41]	rs9939609	Mediterranean diet adherence	WHR	Significant interaction reported	NR	P_int_: 0.023

**Table 4:** Evidence map of PPARG genotype–diet interactions: dietary therapy response and fat-quality modulation. The table summarises each study by variant and dietary exposure/contrast, including dietary therapy and fat quality comparisons, outcome(s), and the direction of the genotype × exposure interaction; effect estimates and interaction *p*-values are reported when available (NR, not reported; NA, not applicable).

Study (Year)	Variant	Exposure (Definition/Threshold)	Outcome(s)	Key Interaction Finding (Direction)	Effect Size/Estimate (If Reported)	*p*-Value
Valeeva et al., 2022 [13]	rs1801282	Diet therapy	Weight loss	CC homozygotes had greater weight loss vs. CG/GG	−2.92 ± 0.57% vs. −0.33 ± 0.70%	*p* (reported): 0.013
Valeeva et al., 2022 [13]	rs1801282	Diet therapy	Waist/hip ratio	CC homozygotes had greater decrease vs. CG/GG	−2.78 ± 0.97% vs. +0.70 ± 1.52%	*p* (reported): 0.05
Maciejewska-Skrendo et al., 2022 [5]	Pro12Ala	PUFA vs. SFA (reviewed evidence)	BMI	12Ala carriers: lower BMI on high PUFA; higher BMI on high SFA	NR	NA (review; varies)

**Table 6:** Evidence map of lipid handling and postprandial phenotype genotype–diet interactions. The table summarises each study by variant/marker; dietary exposure/contrast (including dietary patterns and fat quality or fatty acid exposures); lipid-related outcome(s) (e.g., fasting lipids, postprandial lipaemia, fatty acid profiles, or lipid-linked metabolites); and the direction of the genotype × exposure interaction; effect estimates and interaction *p*-values are reported when available (NR, not reported; NA, not applicable; Pint, interaction *p*-value).

Study (Year)	Variant/Marker/Score	Exposure (Definition/Threshold)	Outcome(s)	Key Interaction Finding (Direction)	Effect Size/Estimate (If Reported)	*p*-Value (Interaction When Applicable)
Primo et al., 2024 [10]	ADIPOQ rs822393	High-fat hypocaloric Mediterranean-pattern diet	HDL-C	Non-T-allele carriers (CC) showed greater HDL-C increase vs. T-allele carriers	+8.9 ± 1.1 vs. +1.7 ± 0.8 mg/dL	*p* = 0.02
Primo et al., 2024 [10]	ADIPOQ rs822393	High-fat hypocaloric Mediterranean-pattern diet	LDL-C	LDL-C reduction occurred, with no clear genotype-dependent difference	NR (similar reduction across genotypes)	*p* = 0.41
De Luis et al., 2021 [9]	APOA5 rs662799	Mediterranean-pattern hypocaloric diet	Triglycerides	Non-C carriers had larger TG reduction than C carriers	−19.3 ± 4.2 vs. −3.2 ± 3.1 mg/dL	*p* = 0.02
Madhu et al., 2022 [48]	TCF7L2 rs7903146	Standardized oral fat challenge	4 h TG; TG AUC (postprandial lipemia)	T-allele carriers showed higher postprandial TG excursions and AUC	NR	*p* < 0.01
Parnell et al., 2025 [11]	TCF7L2 rs7903146	Mediterranean vs. low-fat diet (crossover)	Fatty-acid profile change (Δ-SFA; Δ-MUFA)	Coordinated fatty-acid response was genotype-dependent (CC-directed signal in Mediterranean arm)	NR	p_int(Δ-SFA)_ = 0.0046; p_int(Δ-MUFA)_ = 0.0078
Zhuang et al., 2022 [26]	DPA-associated alleles	*n*-3 PUFA intake	T2D risk (lipid-related pathway signal)	Inverse association between *n*-3 PUFA intake and T2D risk was stronger in participants carrying more DPA-associated alleles	NR	p_int_ = 0.007
Sevilla-González et al., 2024 [15]	SLC16A11 risk haplotype	Lifestyle intervention; higher PUFA exposure	Methylmalonylcarnitine (lipotoxicity-related metabolite)	Higher PUFA exposure was inversely associated with methylmalonylcarnitine in risk-haplotype carriers	β = −0.038	*p* = 0.017

**Table 7:** Evidence map of genotype/score × diet interactions for glycaemic and insulin-signalling outcomes. The table summarises each study by variant or score (GRS/PGS/PRS where applicable); dietary exposure/contrast (macronutrient composition, food group exposures, dietary patterns, or lifestyle interventions); glycaemic/insulin-related outcome(s) (e.g., fasting glucose, insulin resistance indices, β-cell function measures, HbA1c, or diabetes incidence); and the direction of the genotype/score × exposure interaction; effect estimates and interaction *p*-values are reported when available (GRS, genetic risk score; PGS, polygenic score; PRS, polygenic risk score; NR, not reported; NA, not applicable; Pint, interaction *p*-value).

Study (Year)	Variant/Marker/Score	Exposure (Definition/Threshold)	Outcome(s)	Key Interaction Finding (Direction)	Effect Size/Estimate (If Reported)	*p*-Value (Interaction When Applicable)
Hosseinpour-Niazi et al., 2022 [51]	TCF7L2 variants (incl. rs7903146)	Fatty acids, macronutrients, Mediterranean-style diet (reviewed evidence)	Glucose, insulin, HOMA-IR, HOMA-β	Risk-carrier glycaemic responses less favourable with higher SFA; more favourable patterns with unsaturated fat/Mediterranean-style exposures (heterogeneous evidence)	NR	NA (review; varies)
Bauer et al., 2021 [24]	TCF7L2 rs7901695	Carbohydrate intake thresholds (≤48% vs. >48% energy)	VAT, HbA1c, CIR30	Lower carbohydrates linked to less favourable central adiposity/glycaemic phenotypes in CC carriers; higher carbohydrates linked to lower HbA1c and stronger CIR30 in TT carriers	NR	NR
López-Portillo et al., 2021 [33]	16-SNP T2D GRSw (incl. TCF7L2)/TCF7L2 rs7903146	Sugar-sweetened beverage (SSB) intake	Fasting glucose	Positive association of SSB intake with fasting glucose was stronger at higher aggregate genetic risk (graded amplification)	NR	NR
Tolonen et al., 2025 [23]	76-variant T2D GRS	Lifestyle intervention (dietary pattern change over 3 years)	T2D incidence	In high genetic risk, least healthy dietary changes increased T2D risk; healthiest changes reduced risk; minimal effect in low-risk group	OR 3.69 (least healthy change, high risk); OR 0.53 (healthiest change, high risk)	NR
Tieu et al., 2024 [57]	50-SNP T2D PRS	Healthy lifestyle score (postpartum)	Glycaemic abnormalities (5-year postpartum)	High lifestyle score associated with lower glycaemic risk, strongest in highest PRS tertile; null effects in lower-risk tertiles	OR 0.24 (highest PRS tertile)	NR
Mutch et al., 2022 [84]	SCD rs3071 (CC highlighted)	Dietary oils varying in SFA/MUFA (control vs. canola/high-oleic canola)	Fasting glucose	In rs3071 CC carriers, SFA-rich control oil associated with higher fasting glucose; MUFA-rich oils associated with reduction	+0.14 mmol/L under control (SFA-rich) condition (reported in text)	*p* = 0.005
Sevilla-González et al., 2024 [15]	SLC16A11 risk haplotype	Lifestyle intervention; higher PUFA exposure	Methylmalonylcarnitine	Higher PUFA exposure inversely associated with methylmalonylcarnitine in risk-haplotype carriers	β = −0.038	*p* = 0.017
Farrell et al., 2021 [78]	AMY1 copy number variation	Habitual starch intake; controlled starch challenges	Fasting glucose; postprandial glucose/insulin	AMY1 copy number interacted with starch exposure to modify glucose homeostasis	NR	NR
Westerman et al., 2021 [25]	Loci near TRPM2/TRPM3 (interaction signals)	Carbohydrate-containing food groups (GWIS dietary traits/patterns)	HbA1c	Genome-wide interaction signals implicated loci near TRPM2/TRPM3 in HbA1c modification by carbohydrate-containing foods	NR	NR
Chen et al., 2021 [43]	159-SNP adiposity PGS (WHRonly+)	Dietary protein intake	Fasting glucose; β-cell compensation (HOMA-B)	WHRonly+ PGS significantly modified glycaemic response to dietary protein; pathway signal stronger for β-cell compensation than for insulin resistance in narrative synthesis	NR	p_int_ = 0.0007
Billings et al., 2024 [64]	Partitioned T2D polygenic score (β-cell burden)	Lifestyle vs. metformin vs. placebo (DPP)	β-cell function decline	β-cell-risk score predicted declining function independent of intervention allocation (predictive; not a clear diet-specific interaction)	NR	NR

**Table 8:** Evidence map of genotype/score × diet interactions for inflammatory, adipokine, and oxidative stress biomarker outcomes. The table summarises each study by variant or score; dietary exposure/contrast (e.g., dietary patterns, dietary acid load, antioxidant capacity, or nutrient-related exposures); biomarker outcome(s) (inflammatory markers, adipokines, or oxidative stress indicators); and the direction of the genotype/score × exposure interaction; effect estimates and interaction *p*-values are reported when available (NR, not reported; NA, not applicable; Pint, interaction *p*-value).

Study (Year)	Variant/Marker/Score	Exposure (Definition/Threshold)	Outcome(s)	Key Interaction Finding (Direction)	Effect Size/Estimate (If Reported)	*p*-Value (Interaction When Applicable)
Abaj et al., 2022 [35]	APOA2 −265T>C (rs5082)	High dietary acid load (PRAL)	hs-CRP, leptin, ghrelin	In T2D, C-allele carriers with high PRAL had higher hs-CRP, leptin, and ghrelin; no association in T-allele carriers	NR	p_int_ = 0.04
Jafari Azad et al., 2022 [102]	APOA2 −265T>C (rs5082)	Dietary total antioxidant capacity (DTAC)	hs-CRP, SOD, IL-18, PGF2α	Direction differed by genotype: in T carriers, higher DTAC associated with lower hs-CRP and higher SOD; in CC homozygotes, higher DTAC associated with higher IL-18 and PGF2α	NR	*p* = 0.037
Primo et al., 2024 [10]	ADIPOQ rs822393	Hypocaloric Mediterranean-pattern intervention	Adiponectin (plus HDL-C/insulin sensitivity context)	CC (non-T) carriers showed greater adiponectin response and more favourable metabolic improvement than T-allele carriers	NR	NR
Choi et al., 2023 [103]	Antioxidant defence pathway PRS (e.g., GSTA5 and GPX1)	Dietary antioxidants/vitamin C intake	T2D risk (oxidative-stress pathway context)	Higher antioxidant-related dietary exposures attenuated T2D risk associated with higher genetic burden	NR	NR
Hosseinpour-Niazi et al., 2022 [105]	TCF7L2 rs7903146	Diet–inflammation context (trial setting examined)	Inflammatory biomarkers	No genotype-dependent differences in diet-related inflammatory biomarker changes were observed	NR	NS (qualitative)
Hosseinpour-Niazi et al., 2022 [105]	TCF7L2 rs7903146	Legume-based DASH vs. standard DASH	hs-CRP, TNF-α, IL-6	Legume-based DASH reduced inflammatory markers irrespective of genotype	NR	NR

## Data Availability

No new data were created or analyzed in this study. Data sharing is not applicable to this article.

## Supplementary Materials

The following are available online at https://www.mdpi.com/article/10.3390/nu18050815/s1, Table S1: Adiposity & Energy Balance—Included studies. Table S2: Lipid Profile & Fatty-Acid Metabolism—Included studies, Table S3: Insulin/Glucose Signalling—Included studies.

## Abbreviations

The following abbreviations are used in this manuscript:

ADA	American Diabetes Association
APOA2	apolipoprotein A2
APOA5	apolipoprotein A5
APOB	apolipoprotein B
APOE	apolipoprotein E
AUC	area under the curve
BDNF	Brain-derived neurotrophic factor
BMI	Body mass index
CETP	Cholesteryl ester transfer protein
CI	Confidence interval
CIR30	Corrected insulin response at 30 min
CREBRF	cAMP-responsive element binding protein 3 regulatory factor
DASH	Dietary Approaches to Stop Hypertension
DII	Dietary inflammatory index
DPA	docosapentaenoic acid
DTAC	dietary total antioxidant capacity
FADS	fatty acid desaturase genes
FFQs	food frequency questionnaires
FPG	fasting plasma glucose
FTO	fat mass and obesity-associated protein
GI	glycaemic index
GL	glycaemic load
GRS	genetic risk score
HbA1c	glycated haemoglobin
HDL-C	high-density lipoprotein cholesterol
HOMA-B	homeostatic model assessment of β-cell function
HOMA-IR	homeostatic model assessment for insulin resistance
hs-CRP	high-sensitivity C-reactive protein
IL-6	interleukin (IL)-6
IL-18	interleukin (IL)-18
LPL	lipoprotein lipase
LDL-C	low-density lipoprotein cholesterol
MC4R	melanocortin 4 receptor
MTNR1B	melatonin receptor 1B
MUFA	monounsaturated fatty acid
*n*-3 PUFA	omega-3 polyunsaturated fatty acid
OGTT	oral glucose tolerance test
OR	odds ratio
PGS	polygenic score
PPARG	peroxisome proliferator-activated receptor gamma
PRAL	potential renal acid load
PRISMA	Preferred Reporting Items for Systematic Reviews and Meta-Analyses
PRS	polygenic risk score
PREDIMED	Prevención con Dieta Mediterránea
PUFA/PUFAs	polyunsaturated fatty acid(s)
RCT(s)	randomized controlled trial(s)
SAT	subcutaneous adipose tissue
SFA/SFAs	saturated fatty acid(s)
SLC16A11	solute carrier family 16 member 11
SNP(s)	single nucleotide polymorphism(s)
T2D	type 2 diabetes
TCF7L2	transcription factor 7-like 2
TG	triglyceride
TMEM18	transmembrane protein 18
TNF-α	tumour necrosis factor (TNF)-α
VAT	visceral adipose tissue
VDR	vitamin D receptor
VLDL-TGs	very-low-density lipoprotein triglycerides
WC	waist circumference
WHO	World Health Organization
WHR	waist-to-hip ratio
WHtR	waist-to-height ratio
