# Supplementary material for: Personalised Nutrition in Obesity and Prediabetes: Do Genotypes Matter?
Source: Nutrients. 2026 Mar 2;18(5):815. doi: 10.3390/nu18050815 (PMC12987140; doi:10.3390/nu18050815)
Supplement: Supplementary file 1 [file nutrients-18-00815-s001.zip › nutrients-4138404-supplementary.pdf]

Table S1. Adiposity & Energy Balance — Included studies.

Row ID	Study	Title	DOI	Full text retrieved?	Study Type	Population	Sample Size	Genetic Factors	Dietary Factors
S1-01	Tieu et al. - 2024	Genetic risk of type 2 diabetes modifies the association between lifestyle and glycemic health at 5 years postpartum among high-risk women	10.1136/bmjdrc-2023-003942	Yes	RCT	Finnish women, postpartum	314	50-SNP T2D PRS	Healthy lifestyle score
S1-02	Valeeva et al. - 2022	Association of gene polymorphisms with body weight changes in prediabetic patients	10.1007/s11033-022-07254-y	Yes	Intervention	Russian prediabetic women	81	MC4R, PPARG, TCF7L2	Diet ± metformin
S1-03	Sevilla-gonzález et al. - 2024	Metabolomic Profile Alterations Associated with the SLC16A11 Risk Haplotype Following a Lifestyle Intervention in People With Prediabetes	10.1016/j.cdnut.2024.104444	Yes	Quasi-experimental	Mexican-mestizo, prediabetes	52	SLC16A11 haplotype	24-week lifestyle intervention
S1-04	Primo et al. - 2024	Impact of the rs822393 Variant on Adiponectin Levels and Metabolic Parameters after Weight Loss Secondary to a High-Fat Hypocaloric diet with Mediterranean Pattern	10.1159/000539056	Yes	Intervention	Spanish Caucasian, obesity	283	ADIPOQ rs822393	Mediterranean hypocaloric diet
S1-05	Pramono et al. - 2021	The association between vitamin D receptor polymorphisms and tissue-specific insulin resistance in human obesity	10.1038/s41366-021-00744-2	Yes	RCT	European, obesity	553	VDR poly-morphisms	Low-calorie diet + maintenance
S1-06	Huang et al. - 2021	Interaction of Diet/Lifestyle Intervention and TCF7L2 Genotype on Glycemic Control and Adiposity among Overweight or Obese Adults: Big Data from Seven Randomized Controlled Trials Worldwide	10.34133/2021/9897048	Yes	Meta-analysis of RCTs	Overweight/obese adults	4,114	TCF7L2 rs7903146	Diet/lifestyle interventions
S1-07	Billings et al. - 2024	Increased Genetic Risk for β-Cell Failure Is Associated With β-Cell Function Decline in People With Prediabetes	10.2337/db23-0761	Yes	RCT	US, prediabetes	2,647	Partitioned T2D pPS	Lifestyle/metformin/placebo
S1-08	De Soysa et al. - 2021	The fat mass and obesity-associated (FTO) gene allele rs9939609 and glucose tolerance, hepatic and total insulin sensitivity, in adults with obesity	10.1371/journal.pone.0248247	Yes	Cross-sectional	Norwegian, severe obesity	97	FTO rs9939609	Standardized meal test
S1-09	González-salazar et al. - 2022	Effect of the <i>BCAT2</i> polymorphism (rs11548193) on plasma branched-chain amino acid concentrations after dietary intervention in subjects with obesity and insulin resistance	10.1017/S0007114521002920	Yes	Prospective cohort	Mexican, obesity with IR	82	BCAT2 rs11548193	Energy-restricted diet

Row ID	Study	Title	DOI	Full text retrieved?	Study Type	Population	Sample Size	Genetic Factors	Dietary Factors
S1-10	Deluis et al. - 2021	APOA-5 Genetic Variant rs662799: Role in Lipid Changes and Insulin Resistance after a Mediterranean Diet in Caucasian Obese Subjects	10.1155/2021/1257145	Yes	Prospective cohort	Spanish Caucasian, obesity	363	APOA5 rs662799	Mediterranean hypocaloric diet
S1-11	Bauer et al. - 2021	Dietary Macronutrient Intake May Influence the Effects of TCF7L2 rs7901695 Genetic Variants on Glucose Homeostasis and Obesity-Related Parameters: A Cross-Sectional Population-Based Study	10.3390/nu13061936	Yes	Cross-sectional	Polish Caucasian	810	TCF7L2 rs7901695	Macronutrient intake analysis
S1-12	Adamska-patruno et al. - 2021	An Association between Diet and MC4R Genetic Polymorphism, in Relation to Obesity and Metabolic Parameters—A Cross Sectional Population-Based Study	10.3390/ijms222112044	Yes	Cross-sectional	Polish Caucasian	819	MC4R SNPs	Macronutrient intake analysis
S1-13	Wuni et al. - 2022	Impact of Lipid Genetic Risk Score and Saturated Fatty Acid Intake on Central Obesity in an Asian Indian Population	10.3390/nu14132713	Yes	Cross-sectional	Asian Indian	497	CETP/LPL GRS	SFA intake
S1-14	Tolonen et al. - 2025	Changes in food choices and dietary patterns during the lifestyle intervention and their association with type 2 diabetes risk in participants with high or low genetic risk for type 2 diabetes	10.1007/s00394-025-03791-x	Yes	Intervention	Finnish men, prediabetes	883	76-SNP T2D GRS	Group lifestyle intervention
S1-15	Sekar et al. - 2025	High Polyunsaturated Fatty Acid Intake Attenuates the Genetic Risk of Higher Waist Circumference in a Sri Lankan Adult Population	10.3390/nu17172866	Yes	Cross-sectional	Sri Lankan adults	105	10-SNP metabolic GRS	PUFA intake
S1-16	Maciejewska-skrendo et al. - 2022	The Influence of the Differentiation of Genes Encoding Peroxisome Proliferator-Activated Receptors and Their Coactivators on Nutrient and Energy Metabolism	10.3390/nu14245378	Yes	Systematic review	Various populations	Various	PPAR genes	Various interventions
S1-17	Molani-Gol et al. - 2025	The interaction of vitamin D supplementation with Omentin-1 gene polymorphism on metabolic factors and anthropometric indices in women with prediabetes: a study protocol for a double-blind randomized controlled trial	10.1186/s12906-025-05034-2	Yes	RCT	Iranian women, prediabetes	204	Omentin-1 Val109Asp	Vitamin D supplementa-tion
S1-18	Konstantinidou et al. - 2021	Moving forward the Effects of Gene–Diet Interactions on Human Health	10.3390/nu14183782	Yes	Cross-sectional	Chilean adults	2,828	16-SNP T2D GRS	SSB consumption

Row ID	Study	Title	DOI	Full text retrieved?	Study Type	Population	Sample Size	Genetic Factors	Dietary Factors
S1-19	Mutch et al. - 2022	Polymorphisms in the stearoyl-CoA desaturase gene modify blood glucose response to dietary oils varying in MUFA content in adults with obesity	10.1017/S0007114521001264	Yes	RCT crossover	North American, obesity	101	SCD gene SNPs	Oils varying in SFA/MUFA
S1-20	Chen et al. - 2021	Distinct genetic subtypes of adiposity and glycemic changes in response to weight-loss diet intervention: the POUNDS Lost trial	10.1007/s00394-020-02244-x	Yes	RCT	US, over-weight/obese	583	159-SNP adiposity PGS	Reduced-calorie diets
S1-21	Bineid et al. - 2024	A Systematic Review of the Effect of Gene–Lifestyle Interactions on Metabolic-Disease-Related Traits in South Asian Populations	10.1093/nutrit/nuae115	Yes	Systematic review	South Asian populations	109-16,157	Multiple GRS	Diet/physical activity
S1-22	Antwi et al. - 2023	Precision Nutrition to Improve Risk Factors of Obesity and Type 2 Diabetes	10.1007/s13668-023-00491-y	Yes	Narrative review	Various populations	Various	Multiple SNPs/GRS	Precision nutrition
S1-23	Aronica et al. - 2020	Genetic variants for personalised management of very low carbohydrate ketogenic diets	10.1136/bmjnph-2020-000167	Yes	Review	Various populations	Various	Multiple SNPs	Ketogenic diet
S1-24	Alsulami et al. - 2021	Lower Dietary Intake of Plant Protein Is Associated with Genetic Risk of Diabetes-Related Traits in Urban Asian Indian Adults	10.3390/nu13093064	Yes	Cross-sectional	Asian Indian	1,062	TCF7L2/FTO GRS	Plant protein intake
S1-25	Abaj et al. - 2021	Are caveolin-1 minor alleles more likely to be risk alleles in insulin resistance mechanisms in metabolic diseases?	10.1186/s13104-021-05597-6	Yes	Cross-sectional	Iranian women, over-weight/obese	404	CAV1 rs3807992	Dietary assessment
S1-26	Yamamoto et al. - 2025	The <i>SELENOP</i> Polymorphism rs7579 Predicts Hepatic Steatosis in Females With Insulin Resistance in the General Population	10.1210/jendso/bvaf144	Yes	Cross-sectional	Japanese adults	900	SELENOP rs7579	Not specified
S1-27	Sepulveda-villegas et al. - 2025	The fat-mass and obesity-associated gene rs9939609 T allele is prominent among the native Mexican population and is associated with risk for Type 2 diabetes and metabolic dysfunction-associated steatotic liver disease	10.3389/fnut.2025.1569342	Yes	Cross-sectional	West Mexican populations	684	FTO rs9939609	Three-day dietary record
S1-28	Sekar et al. - 2024	A Novel Interaction between a 23-SNP Genetic Risk Score and Monounsaturated Fatty Acid Intake on HbA1c Levels in Southeast Asian Women	10.3390/nu16173022	Yes	Cross-sectional	Indonesian women	106	23-SNP GRS	MUFA intake

Row ID	Study	Title	DOI	Full text retrieved?	Study Type	Population	Sample Size	Genetic Factors	Dietary Factors
S1-29	Parnell et al. - 2025	CC Genotype at TCF7L2 Diabetes Risk Locus rs7903146 Directs a Coordinated Fatty Acid Response to a Mediterranean Diet Intervention: A Randomized Controlled Trial	10.1159/000542468	Yes	RCT crossover	US adults	35	TCF7L2 rs7903146	Mediterranean vs low-fat diet
S1-30	Mostad et al. - 2023	NEFA Dynamics in Adults With Severe Obesity and Insulin Resistance: No Coupling to the rs9939609 <i>FTO</i> Risk Allele	10.1210/jendso/bvad101	Yes	Cross-sectional	Norwegian, severe obesity	97	FTO rs9939609	Not applicable
S1-31	Ezgi Ozen et al. - 2024	Association between body composition and cardiometabolic disease risk: role of dietary fat intake and APOLIPOPROTEIN E genotype on this relationship	10.1017/S0029665124000053	Yes	Narrative review	Various populations	Various	APOE genotype	SFA vs UFA replacement
S1-32	Farrell et al. - 2021	Effect of AMY1 copy number variation and various doses of starch intake on glucose homeostasis: data from a cross-sectional observational study and a crossover meal study	10.1186/s12263-021-00701-8	Yes	Cross-sectional + meal study	Swedish adults	1,764	AMY1 CNV	Starch intake
S1-33	Górczyńska-kosiorz et al. - 2024	Bone Mineral Density and the Risk of Type-2 Diabetes in Postmenopausal Women: rs4988235 Polymorphism Associated with Lactose Intolerance Effects	10.3390/nu16173002	Yes	Retrospective cohort	Polish post-menopausal women	607	MCM6 rs4988235	Calcium/dairy intake
S1-34	Alanazi et al. - 2024	A Systematic Review of the Gene–Lifestyle Interactions on Metabolic Disease-Related Outcomes in Arab Populations	10.3390/nu16152519	Yes	Systematic review	Arab populations	Various	FTO, TCF7L2, MC4R, MTHFR	Diet/physical activity
S1-35	Al-odinan et al. - 2025	Interaction between the TCF7L2 gene and dietary intake on metabolic syndrome risk factors among Saudi Arabian adults	10.3389/fnut.2025.1513088	Yes	Cross-sectional	Saudi Arabian adults	271	TCF7L2 rs7903146	Energy/macronutrient intake
S1-36	Li et al. - 2025	Marine N-3 Fatty Acids Mitigate Hyperglycemia in Prediabetes by Improving Muscular Glucose Transporter 4 Translocation and Glucose Homeostasis	10.34133/research.0683	Yes	Prospective cohort	UK Biobank, prediabetes	48,358	GLUT4/GCKR/FADS1 SNPs	Fish oil supplementation
S1-37	Madhu et al. - 2022	TCF7L2 gene associated postprandial triglyceride dysmetabolism- a novel mechanism for diabetes risk among Asian Indians	10.3389/fendo.2022.973718	Yes	Case-control	Asian Indian	620	TCF7L2 rs7903146	Standardized fat challenge

Row ID	Study	Title	DOI	Full text retrieved?	Study Type	Population	Sample Size	Genetic Factors	Dietary Factors
S1-38	Vimaleswaran et al. - 2021	GeNuIne (gene–nutrient interactions) Collaboration: towards implementing multi-ethnic population-based nutrigenetic studies of vitamin B₁₂ and D deficiencies and metabolic diseases	10.1017/S0029665121002822	Yes	Multi-population study	Multi-ethnic populations	Various	Multiple GRS	Macronutrient intake
S1-39	Patarrão et al. - 2022	Prediabetes blunts DPP4 genetic control of postprandial glycaemia and insulin secretion	10.1007/s00125-021-05638-6	Yes	Cohort + experimental	Portuguese + mouse models	1,084	DPP4 SNPs	High-fat diet (mice)
S1-40	Mirrakhimov et al. - 2024	Association of leptin receptor gene Gln223Arg polymorphism with insulin resistance and hyperglycemia in patients with metabolic syndrome	10.5114/aoms/170121	Yes	Cross-sectional	Kyrgyz population	237	LEPR Gln223Arg	Not specified
S1-41	Mojsak et al. - 2021	A Preliminary Study Showing the Impact of Genetic and Dietary Factors on GC–MS-Based Plasma Metabolome of Patients with and without PROX1-Genetic Predisposition to T2DM up to 5 Years Prior to Prediabetes Appearance	10.3390/cimb43020039	Yes	Meal challenge study	Polish Caucasian men	18	PROX1 rs340874	HC vs NC meal
S1-42	Padilla-martinez et al. - 2022	Testing the Utility of Polygenic Risk Scores for Type 2 Diabetes and Obesity in Predicting Metabolic Changes in a Prediabetic Population: An Observational Study	10.3390/ijms232416081	Yes	Prospective cohort	Polish, prediabetes	446	T2D PRS (68 SNPs), Obesity PRS (21 SNPs)	Not specified
S1-43	Burden et al. - 2021	The CREBRF diabetes-protective rs373863828-A allele is associated with enhanced early insulin release in men of Māori and Pacific ancestry	10.1007/s00125-021-05552-x	Yes	Cross-sectional	Māori/Pacific men	172	CREBRF rs373863828	Mixed meal tolerance test
S1-44	Alathari et al. - 2022	Interactions between Vitamin D Genetic Risk and Dietary Factors on Metabolic Disease-Related Outcomes in Ghanaian Adults	10.3390/nu14132763	Yes	Cross-sectional	Ghanaian adults	302	Vitamin D-related GRS	Fiber/fat intake
S1-45	Zhuang et al. - 2022	Circulating Fatty Acids and Genetic Predisposition to Type 2 Diabetes: Gene-Nutrient Interaction Analysis	10.2337/dc21-2048	Yes	Prospective cohort	UK Biobank	95,854	Pathway-specific GRS	Plasma fatty acids
S1-46	Rhee et al. - 2024	Discrepancy Between Genetically Predicted and Observed BMI Predicts Incident Type 2 Diabetes	10.2337/dc24-0879	Yes	Prospective cohort	UK Biobank + Korean	339,584	BMI PRS	Not specified

Row ID	Study	Title	DOI	Full text retrieved?	Study Type	Population	Sample Size	Genetic Factors	Dietary Factors
S1-47	Sørensen et al. - 2022	Do gene–environment interactions have implications for the precision prevention of type 2 diabetes?	10.1007/s00125-021-05639-5	Yes	Review	Various populations	Various	FTO locus, PRS	Behavioral modifications
S1-48	Mongkolsucharitkul et al. - 2024	Metabolic and genetic risk factors associated with pre-diabetes and type 2 diabetes in Thai healthcare employees: A long-term study from the Siriraj Health (SIH) cohort study	10.1371/journal.pone.0303085	Yes	Prospective cohort	Thai healthcare workers	5,011	TCF7L2 SNPs	Not specified
S1-49	Hosseinpour-niazi et al. - 2022	Effect of TCF7L2 on the relationship between lifestyle factors and glycemic parameters: a systematic review	10.1186/s12937-022-00813-w	Yes	Systematic review	Various populations	Various	TCF7L2 variants	Various interventions
S1-50	Jiang et al. - 2024	Maternal smoking, nutritional factors at different life stage, and the risk of incident type 2 diabetes: a prospective study of the UK Biobank	10.1186/s12916-024-03256-8	Yes	Prospective cohort	UK Biobank	460,234	Pathway-specific PRS	AHEI/DII dietary patterns
S1-51	Huang et al. - 2021	Interaction of Diet/Lifestyle Intervention and TCF7L2 Genotype on Glycemic Control and Adiposity among Overweight or Obese Adults: Big Data from Seven Randomized Controlled Trials Worldwide	10.34133/2021/9897048	Yes	Prospective cohort	Chinese adults	93,488	T2D PRS	Healthy diet assessment
S1-52	Jang et al. - 2025	<i>SLC30A8</i> Rare Variant Modify Contribution of Common Genetic and Lifestyle Factors toward Type 2 Diabetes Mellitus	10.4093/dmj.2024.0830	Yes	Prospective cohort	Korean adults	146,284	SLC30A8 I349F, T2D PRS	Healthy lifestyle score
S1-53	Gurtan et al. - 2022	Analyzing human knockouts to validate GPR151 as a therapeutic target for reduction of body mass index	10.1371/journal.pgen.1010093	Yes	Cross-sectional + experimental	Pakistani + mice	30,833	GPR151 variants	Standard vs high-fat diet
S1-54	Ajabnoor et al. - 2022	ABCA1 C69T Gene Polymorphism Association with Dysglycemia in Saudi Prediabetic Adults	10.3390/genes13122277	Yes	Cross-sectional	Saudi adults	650	ABCA1 rs1800977	Not specified
S1-55	Williamson et al. - 2023	Genome-wide association study and functional characterization identifies candidate genes for insulin-stimulated glucose uptake	10.1038/s41588-023-01408-9	Yes	GWAS	Multi-ancestry	>55,000	Novel insulin-related SNPs	Not applicable
S1-56	Luukkonen et al. - 2021	The PNPLA3-I148M Variant Confers an Antiatherogenic Lipid Profile in Insulin-resistant Patients	10.1210/clinem/dgaa729	Yes	Cross-sectional	Finnish, obesity	643	PNPLA3-I148M	Not applicable

Row ID	Study	Title	DOI	Full text retrieved?	Study Type	Population	Sample Size	Genetic Factors	Dietary Factors
S1-57	Lam et al. - 2025	Integrative Roles of Functional Foods, Microbiotics, Nutrigenetics, and Nutrigenomics in Managing Type 2 Diabetes and Obesity	10.3390/nu17040608	Yes	Systematic review	Various populations	Various	FTO, PPARγ, ADIPOQ, TCF7L2	Mediterranean diet
S1-58	Binjawhar et al. - 2023	Genetic Variants of HNF4A, WFS1, DUSP9, FTO, and ZFAND6 Genes Are Associated with Prediabetes Susceptibility and Inflammatory Markers in the Saudi Arabian Population	10.3390/genes14030536	Yes	Cross-sectional	Saudi adults	1,129	HNF4A, WFS1, DUSP9, FTO, ZFAND6	Not specified

Table S2. Lipid Profile & Fatty-Acid Metabolism — Included studies.

Row ID	Study	Title	DOI	Full text retrieved?	Study Type	Population (n; status)	Key Genetic Variants	Fat Quality Exposure	Primary Outcome Domain
S2-01	Tolonen et al. - 2025	Changes in food choices and dietary patterns during the lifestyle intervention and their association with type 2 diabetes risk in participants with high or low genetic risk for type 2 diabetes	10.1007/s00394-025-03791-x	Yes	Non-randomized intervention	n=883; men with prediabetes, BMI ≥25	76-variant T2D GRS	Not specified	T2D incidence, dietary patterns
S2-02	Primo et al. - 2024	Impact of the rs822393 Variant on Adiponectin Levels and Metabolic Parameters after Weight Loss Secondary to a High-Fat Hypocaloric diet with Mediterranean Pattern	10.1159/000539056	Yes	Dietary intervention trial	n=283; obese, mean BMI 36.0	ADIPOQ rs822393	Mediterranean-pattern high-fat hypocaloric diet (60% MUFA, 30% SFA, 10% PUFA)	HDL-C, adiponectin, lipid profile
S2-03	Deluis et al. - 2021	APOA-5 Genetic Variant rs662799: Role in Lipid Changes and Insulin Resistance after a Mediterranean Diet in Caucasian Obese Subjects	10.1155/2021/1257145	Yes	Observational cohort with intervention	n=363; Caucasian obese, mean BMI 36.2	APOA5 rs662799	Mediterranean-pattern hypocaloric diet (55% MUFA, 30% SFA, 15% PUFA)	Triglycerides, insulin, HOMA-IR
S2-04	Sevilla-gonzález et al. - 2024	Metabolomic Profile Alterations Associated with the SLC16A11 Risk Haplotype Following a Lifestyle Intervention in People With Prediabetes	10.1016/j.cdnut.2024.104444	Yes	Quasiexperimental	n=52; Mexican mestizo, prediabetes, mean BMI 30.0	SLC16A11 risk haplotype	Hypocaloric diet with 30% lipids; PUFA intake assessed	Metabolomic profile, lipid metabolites
S2-05	Mutch et al. - 2022	Polymorphisms in the stearoyl-CoA desaturase gene modify blood glucose response to dietary oils varying in MUFA content in adults with obesity	10.1017/S0007114521001264	Yes	Randomized crossover RCT	n=101; abdominal obesity, prediabetes criteria	SCD rs1502593, rs3071, rs522951	Control oil (36.6% SFA/28.2% MUFA), canola oil (6.2% SFA/63.1% MUFA), high-oleic canola (5.8% SFA/74.7% MUFA)	Blood glucose (primary), gene-oil interactions
S2-06	Parnell et al. - 2025	CC Genotype at TCF7L2 Diabetes Risk Locus rs7903146 Directs a Coordinated Fatty Acid Response to a Mediterranean Diet Intervention: A Randomized Controlled Trial	10.1159/000542468	Yes	Randomized controlled trial	n=35; over-weight/obese, mean BMI 30.3	TCF7L2 rs7903146	Mediterranean diet (41% fat, high MUFA/PUFA) vs. low-fat diet (30% fat)	Fatty acid composition, delta-SFA, delta-MUFA
S2-07	Li et al. - 2025	Marine N-3 Fatty Acids Mitigate Hyperglycemia in Prediabetes by Improving Muscular Glucose Transporter 4 Translocation and Glucose Homeostasis	10.34133/research.0683	Yes	Observational cohort	n=48,358; prediabetes, mean BMI 28.9	GCKR rs780094, FADS1 rs174555, GLUT4 rs5435/rs8082645	DHA and EPA (fish oil supplementa-tion)	T2D risk, n-3 PUFA levels, n-6/n-3 ratio
S2-08	Pramono et al. - 2021	The association between vitamin D receptor polymorphisms and tissue-specific insulin resistance in human obesity	10.1038/s41366-021-00744-2	Yes	Randomized controlled trial	n=553; obese, mean BMI 34.8	VDR FokI rs10735810, TaqI, ApaI, BsmI	Not specified	Adipose tissue insulin resistance, FFA

Row ID	Study	Title	DOI	Full text retrieved?	Study Type	Population (n; status)	Key Genetic Variants	Fat Quality Exposure	Primary Outcome Domain
S2-09	Sekar et al. - 2025	High Polyunsaturated Fatty Acid Intake Attenuates the Genetic Risk of Higher Waist Circumference in a Sri Lankan Adult Population	10.3390/nu17172866	Yes	Cross-sectional	n=105; Sri Lankan adults, age 25-50	10-SNP metabolic GRS (TCF7L2, CAPN10, FTO, KCNJ11, MC4R)	PUFA intake (≥3.1 g/day)	Waist circum-ference
S2-10	Adamska-patruno et al. - 2021	An Association between Diet and MC4R Genetic Polymorphism, in Relation to Obesity and Metabolic Parameters—A Cross Sectional Population-Based Study	10.3390/ijms222112044	Yes	Cross-sectional	n=819; mixed BMI categories, 50.2% predia-betes/diabetes	MC4R rs17782313, rs12970134, rs633265, rs135034	Not specified	VAT, triglycerides, glucose
S2-11	Zhuang et al. - 2022	Circulating Fatty Acids and Genetic Predisposition to Type 2 Diabetes: Gene-Nutrient Interaction Analysis	10.2337/dc21-2048	Yes	Observational cohort	n=95,854; White British	424-variant T2D PRS; pathway-specific GRS	Circulating SFA, MUFA, PUFA, n-3, n-6 biomarkers	T2D incidence, FA-genetic risk interactions
S2-12	Madhu et al. - 2022	TCF7L2 gene associated postprandial triglyceride dysmetabolism- a novel mechanism for diabetes risk among Asian Indians	10.3389/fendo.2022.973718	Yes	Case-control	n=620; Asian Indians, NGT and T2DM/prediabetes	TCF7L2 rs7903146	Standardized oral fat challenge test	Postprandial triglycerides
S2-13	Hosseinpour-niazi et al. - 2022	Effect of TCF7L2 on the relationship between lifestyle factors and glycemic parameters: a systematic review	10.1186/s12937-022-00813-w	Yes	Systematic review	Multiple populations; n=120-48,000 per study	TCF7L2 multiple SNPs	SFA, total fat, fiber	Glycemic parameters, insulin resistance
S2-14	Alathari et al. - 2022	Interactions between Vitamin D Genetic Risk and Dietary Factors on Metabolic Disease-Related Outcomes in Ghanaian Adults	10.3390/nu14132763	Yes	Cross-sectional	n=302; Ghanaian adults, mean BMI 26.6	Vitamin D-GRS (8 SNPs)	Total fat, SFA, MUFA, PUFA	BMI, HbA1c
S2-15	Wuni et al. - 2022	Impact of Lipid Genetic Risk Score and Saturated Fatty Acid Intake on Central Obesity in an Asian Indian Population	10.3390/nu14132713	Yes	Observational cross-sectional	n=497; Asian Indians, 52% T2D	CETP rs4783961, LPL rs327, rs3200218	SFA intake	Waist circum-ference
S2-16	Sehgal et al. - 2022	Indolepropionic Acid, a Gut Bacteria-Produced Tryptophan Metabolite and the Risk of Type 2 Diabetes and Non-Alcoholic Fatty Liver Disease	10.3390/nu14214695	Yes	Observational cohort	n=522 (IGT); Finnish	TCF7L2, FTO rs9939609, PPARG	Not specified (fiber, IPA focus)	T2D risk, IPA levels
S2-17	Mostad et al. - 2023	NEFA Dynamics in Adults With Severe Obesity and Insulin Resistance: No Coupling to the rs9939609 <i>FTO</i> Risk Allele	10.1210/jendso/bvad101	Yes	Cross-sectional	n=97; severe obesity (BMI ≥35), no diabetes	FTO rs9939609	Not specified	NEFA dynamics

Row ID	Study	Title	DOI	Full text retrieved?	Study Type	Population (n; status)	Key Genetic Variants	Fat Quality Exposure	Primary Outcome Domain
S2-18	Molani-Gol et al. - 2025	The interaction of vitamin D supplementation with Omentin-1 gene polymorphism on metabolic factors and anthropometric indices in women with prediabetes: a study protocol for a double-blind randomized controlled trial	10.1186/s12906-025-05034-2	Yes	Double-blind RCT	n=204; women with prediabetes, 70.8% over-weight/obese	Omentin-1 Val109Asp rs2274907	Not specified (vitamin D supplementa-tion)	HDL-C, metabolic biomarkers
S2-19	Huang et al. - 2021	Interaction of Diet/Lifestyle Intervention and TCF7L2 Genotype on Glycemic Control and Adiposity among Overweight or Obese Adults: Big Data from Seven Randomized Controlled Trials Worldwide	10.34133/2021/9897048	Yes	Meta-analysis of 7 RCTs	n=4,114; over-weight/obese, mean BMI 32.2	TCF7L2 rs7903146	Not specified	Fasting glucose, waist circum-ference
S2-20	González-salazar et al. - 2022	Effect of the <i>BCAT2</i> polymorphism (rs11548193) on plasma branched-chain amino acid concentrations after dietary intervention in subjects with obesity and insulin resistance	10.1017/S0007114521002920	Yes	Prospective cohort	n=82; obese, mean BMI 38.1, HOMA-IR ≥2.5	BCAT2 rs11548193	Energy-restricted diet (not fat-specific)	BCAA, lipid profile
S2-21	Bauer et al. - 2021	Dietary Macronutrient Intake May Influence the Effects of TCF7L2 rs7901695 Genetic Variants on Glucose Homeostasis and Obesity-Related Parameters: A Cross-Sectional Population-Based Study	10.3390/nu13061936	Yes	Cross-sectional	n=810; Caucasian, mixed BMI, 50.2% predia-betes/diabetes	TCF7L2 rs7901695	Not specified (macronutri-ent intake)	VAT, SAT, glucose homeostasis
S2-22	Franzago et al. - 2022	Nutrigenetic variants and response to diet/lifestyle intervention in obese subjects: a pilot study	10.1007/s00592-021-01787-7	Yes	Observational cohort	n=18; over-weight/obese, T2D or dysglycemia	FTO rs9939609, MC4R rs17782313, LPL rs326, NPY rs16147, IRS-1 rs2943641	Mediterranean diet	Weight loss, BMI, total cholesterol
S2-23	Zuaiter et al. - 2025	Adherence to Mediterranean Diet Among Prediabetic Patients in East Jerusalem	10.3390/nu17111777	Yes	Observational cohort	n=172; prediabetic adults, 62.2% obese	Not studied	Mediterranean diet adherence	BMI, waist circumfer-ence
S2-24	Sepulveda-villegas et al. - 2025	The fat-mass and obesity-associated gene rs9939609 T allele is prominent among the native Mexican population and is associated with risk for Type 2 diabetes and metabolic dysfunction-associated steatotic liver disease	10.3389/fnut.2025.1569342	Yes	Cross-sectional	n=684; West Mexican, mean BMI 28.3	FTO rs9939609	Not specified	Triglycerides, VLDL-c, insulin
S2-25	Sekar et al. - 2024	A Novel Interaction between a 23-SNP Genetic Risk Score and Monounsaturated Fatty Acid Intake on HbA1c Levels in Southeast Asian Women	10.3390/nu16173022	Yes	Cross-sectional pilot	n=106; Minangkabau women	23-SNP GRS (incl. TCF7L2, FTO, MC4R)	MUFA intake	HbA1c

Row ID	Study	Title	DOI	Full text retrieved?	Study Type	Population (n; status)	Key Genetic Variants	Fat Quality Exposure	Primary Outcome Domain
S2-26	Tieu et al. - 2024	Genetic risk of type 2 diabetes modifies the association between lifestyle and glycemic health at 5 years postpartum among high-risk women	10.1136/bmjdrc-2023-003942	Yes	Randomized controlled trial	n=314; women post-GDM, mean BMI 31.6	50-allele T2D PRS	Not specified (lifestyle score)	Glycemic abnormalities
S2-27	Valeeva et al. - 2022	Association of gene polymorphisms with body weight changes in prediabetic patients	10.1007/s11033-022-07254-y	Yes	Case-control with intervention	n=81; prediabetic women, Eastern European	MC4R rs17782313, PPARG rs1801282, TCF7L2 rs7903146	Not specified (diet therapy ± metformin)	Weight loss, fat mass
S2-28	Bineid et al. - 2024	A Systematic Review of the Effect of Gene–Lifestyle Interactions on Metabolic-Disease-Related Traits in South Asian Populations	10.1093/nutrit/nuae115	Yes	Cross-sectional	n=1,886; Asian Indians, NGT and T2D	Omentin rs2274907	Not specified	Adiponectin, car-diometabolic health
S2-29	Lima et al. - 2025	Interaction Between Dietary Fiber Intake and MTNR1B rs10830963 Polymorphism on Glycemic Profiles in Young Brazilian Adults	10.3390/genes16050497	Yes	Cross-sectional	n=200; Brazilian young adults, mean age 21.3	MTNR1B rs10830963	Dietary fiber (not fat-specific)	Glycemic markers
S2-30	Oh et al. - 2025	Identification of Novel Genetic Variants and Food Intake Factors Associated with Type 2 Diabetes in South Korean Adults, Using an Illness–Death Model	10.3390/ijms26062597	Yes	Observational cohort	n=4,126; Korean adults	GCK rs4607517, CAMK2B rs758982, and others	Dietary patterns (not fat-specific)	T2D progression
S2-31	Mojsak et al. - 2021	A Preliminary Study Showing the Impact of Genetic and Dietary Factors on GC–MS-Based Plasma Metabolome of Patients with and without PROX1-Genetic Predisposition to T2DM up to 5 Years Prior to Prediabetes Appearance	10.3390/cimb43020039	Yes	Observational cohort	n=18; nondiabetic men, mean BMI ~28.5	PROX1 rs340874	HC and NC meal challenges (not fat-specific)	Plasma metabolome, palmitic acid
S2-32	Mongkolsucharitkul et al. - 2024	Metabolic and genetic risk factors associated with pre-diabetes and type 2 diabetes in Thai healthcare employees: A long-term study from the Siriraj Health (SIH) cohort study	10.1371/journal.pone.0303085	Yes	Observational cohort	n=5,011; Thai healthcare workers, 51% over-weight/obese	TCF7L2 rs7903146, rs4506565	Not specified	T2D and prediabetes prevalence
S2-33	Konstantinidou et al. - 2021	Moving forward the Effects of Gene–Diet Interactions on Human Health	10.3390/nu14183782	Yes	Cross-sectional	n=2,828; Latin American, mean BMI 29.3	16-SNP T2D GRSw (incl. TCF7L2, MTNR1B)	SSB intake (not fat-specific)	Fasting glucose
S2-34	Padilla-martinez et al. - 2022	Testing the Utility of Polygenic Risk Scores for Type 2 Diabetes and Obesity in Predicting Metabolic Changes in a Prediabetic Population: An Observational Study	10.3390/ijms23246081	Yes	Observational cohort	n=446; prediabetic, median BMI 26.9	T2D PRS (68 SNPs), obesity PRS (21 SNPs)	Not specified	Fat mass, 2h glucose

Row ID	Study	Title	DOI	Full text retrieved?	Study Type	Population (n; status)	Key Genetic Variants	Fat Quality Exposure	Primary Outcome Domain
S2-35	Das et al. - 2025	CGMacros: a pilot scientific dataset for personalized nutrition and diet monitoring	10.1038/s41597-025-05851-7	Yes	Observational cohort	n=45; healthy, pre-diabetes, T2D	Not studied	CGM-linked macronutri-ent monitoring	Glucose response
S2-36	Billings et al. - 2024	Increased Genetic Risk for β-Cell Failure Is Associated With β-Cell Function Decline in People With Prediabetes	10.2337/db23-0761	Yes	Randomized controlled trial	n=2,647; prediabetes, mean BMI ~34	Partitioned T2D PPS (β-cell, liver/lipid, obesity, lipodystro-phy, proinsulin)	Not specified (lifestyle vs. metformin vs. placebo)	β-cell function, triglycerides
S2-37	Farrell et al. - 2021	Effect of AMY1 copy number variation and various doses of starch intake on glucose homeostasis: data from a cross-sectional observational study and a crossover meal study	10.1186/s12263-021-00701-8	Yes	Cross-sectional + crossover meal study	n=1,764 (ob-servational); n=19 (meal study)	AMY1 copy number variation	Starch doses (not fat-specific)	Fasting glucose, BMI, postprandial glycemia
S2-38	De Soysa et al. - 2021	The fat mass and obesity-associated (FTO) gene allele rs9939609 and glucose tolerance, hepatic and total insulin sensitivity, in adults with obesity	10.1371/journal.pone.0248247	Yes	Cross-sectional	n=97; obese, mean BMI 42.8	FTO rs9939609	Not specified	Insulin sensitivity, glucose tolerance
S2-39	Fu et al. - 2024	Vitamin D Status, Vitamin D Receptor Polymorphisms, and Risk of Type 2 Diabetes: A Prospective Cohort Study	10.1210/clinem/dgae221	Yes	Prospective cohort	n=379,699; UK Biobank	VDR rs7975232, rs1544410, rs2228570, rs731236	Not specified (vitamin D focus)	T2D incidence, triglyceride mediation
S2-40	Gayathri et al. - 2023	Effect of almond consumption on insulin sensitivity and serum lipids among Asian Indian adults with overweight and obesity– A randomized controlled trial	10.3389/fnut.2022.1055923	Yes	Randomized controlled trial	n=400; overweight Asian Indians, mean BMI 28.4	Not studied	Almonds (high MUFA)	Total cholesterol, triglycerides
S2-41	Cheng et al. - 2022	A randomized controlled trial for response of microbiome network to exercise and diet intervention in patients with nonalcoholic fatty liver disease	10.1038/s41467-022-29968-0	Yes	Randomized controlled trial	n=115; NAFLD + prediabetes, age 50-65	Not studied	Diet arm (10% SFA, 15-20% MUFA, 10% PUFA)	Gut microbiome, liver fat
S2-42	Kerlikowsky et al. - 2025	Effects of 12 Weeks of Calanus Oil Supplementation on Cardiac Diastolic Function in Obese and Prediabetic Women—A Pilot Study	10.3390/metabo15090596	Yes	Proof-of-principle trial	n=20; prediabetic obese women, mean BMI 34	Not studied	Calanus oil (276 mg EPA + 256 mg DHA)	Diastolic function, triglycerides
S2-43	Basiri and Rajanala - 2025	Effects of Individualized Nutrition Therapy and Continuous Glucose Monitoring on Dietary and Sleep Quality in Individuals with Prediabetes and Overweight or Obesity	10.3390/nu17091507	Yes	Randomized controlled trial	n=30; prediabetes, BMI 25-39.9	Not studied	Not specified (CGM-guided nutrition)	Diet quality, sleep

Row ID	Study	Title	DOI	Full text retrieved?	Study Type	Population (n; status)	Key Genetic Variants	Fat Quality Exposure	Primary Outcome Domain
S2-44	Alsulami et al. - 2021	Lower Dietary Intake of Plant Protein Is Associated with Genetic Risk of Diabetes-Related Traits in Urban Asian Indian Adults	10.3390/nu13093064	Yes	Cross-sectional	n=200; Brazilian young adults	10-SNP metabolic GRS (TCF7L2, FTO, MC4R, KCNQ1, others)	Total fat intake	Insulin, HOMA-IR, HOMA-B
S2-45	Al-odinan et al. - 2025	Interaction between the TCF7L2 gene and dietary intake on metabolic syndrome risk factors among Saudi Arabian adults	10.3389/fnut.2025.1513088	Yes	Cross-sectional	n=271; Saudi adults, BMI 18-35	TCF7L2 rs7903146	SFA intake; total energy	Waist circumference; insulin

Table S3. Insulin/Glucose Signalling — Included studies.

Row ID	Study	Title	DOI	Full text retrieved?	Study Type	Population	Sample Size	Genetic Factors	Dietary Factors
S3-01	Tolonen et al. - 2025	Changes in food choices and dietary patterns during the lifestyle intervention and their association with type 2 diabetes risk in participants with high or low genetic risk for type 2 diabetes	10.1007/s00394-025-03791-x	Yes	Intervention (3-year lifestyle)	Caucasian men with prediabetes, BMI ≥25, age 50-75 y	n=883	76-variant GRS for T2D	Healthy vs. T2D<br>unhealthy incidence<br>dietary patterns via<br>FFQ
S3-02	Tieu et al. - 2024	Genetic risk of type 2 diabetes modifies the association between lifestyle and glycemic health at 5 years postpartum among high-risk women	10.1136/bmjdrc-2023-003942	Yes	RCT follow-up (5 y post-partum)	Women with BMI ≥30 and/or prior GDM, mean age 38.7 y	n=314	50-SNP PRS for T2D	Healthy Glycemic<br>Lifestyle abnormali-<br>Score (diet, ties PA,<br>(predia-smoking)<br>betes/diabetes) via<br>FFQ
S3-03	Valeeva et al. - 2022	Association of gene polymorphisms with body weight changes in prediabetic patients	10.1007/s11033-022-07254-y	Yes	Intervention (3-month diet ± metformin)	Eastern European prediabetic women, age ~49 y	n=81	MC4R rs17782313, PPARG rs1801282, TCF7L2 rs7903146	Diet Body therapy<br>weight/composition<br>(55% changes energy<br>from carbs) ±<br>metformin
S3-04	Sevilla-gonzález et al. - 2024	Metabolomic Profile Alterations Associated with the SLC16A11 Risk Haplotype Following a Lifestyle Intervention in People With Prediabetes	10.1016/j.cdnut.2024.104444	Yes	Quasi-experimental (24-wk lifestyle)	Mexican-mestizo with prediabetes, BMI 25-45	n=52	SLC16A11 risk haplotype	Lifestyle in-<br>Metabolomic<br>tervention profile;<br>(45% carbs); postpran-<br>PUFA dial glucose<br>intake assessed
S3-05	Li et al. - 2025	Marine N-3 Fatty Acids Mitigate Hyperglycemia in Prediabetes by Improving Muscular Glucose Transporter 4 Translocation and Glucose Homeostasis	10.34133/research.0683	Yes	Prospective cohort + animal/cell study	Prediabetes, majority White (89.3%)	n=48,358	GCKR rs780094, FADS1 rs174555, GLUT4 rs5435/rs8082645	Fish oil T2D<br>(DHA/EPA) incidence<br>supplemen-tation
S3-06	Primo et al. - 2024	Impact of the rs822393 Variant on Adiponectin Levels and Metabolic Parameters after Weight Loss Secondary to a High-Fat Hypocaloric diet with Mediterranean Pattern	10.1159/000539056	Yes	Intervention (12-wk diet)	Caucasian adults with obesity, BMI >30, age 33-64 y	n=283	ADIPOQ rs822393	High-fat Insulin,<br>hypocaloric HOMA-<br>IR, Mediter-<br>adiponectin ranean<br>diet
S3-07	Mutch et al. - 2022	Polymorphisms in the stearoyl-CoA desaturase gene modify blood glucose response to dietary oils varying in MUFA content in adults with obesity	10.1017/S0007114521001264	Yes	RCT crossover (6-wk per oil)	Obese adults, BMI ~31, age 20-65 y	n=101	SCD rs1502593, rs3071, rs522951	Dietary oils Fasting<br>varying in blood<br>SFA/MUFA glucose<br>(controlled feeding)

Row ID	Study	Title	DOI	Full text retrieved?	Study Type	Population	Sample Size	Genetic Factors	Dietary Factors
S3-08	Chen et al. - 2021	Distinct genetic subtypes of adiposity and glycemic changes in response to weight-loss diet intervention: the POUNDS Lost trial	10.1007/s00394-020-02244-x	Yes	RCT (2-year diet interven-tion)	Overweight/obese adults, BMI 25-40, mostly White	n=692	159-SNP PGS (4 adiposity subtypes)	Diets Fasting varying in glucose, carbohy-insulin, drate HOMA-IR, (35-65% HOMA-B energy) and protein
S3-09	Gkouskou et al. - 2022	Genetically-Guided Medical Nutrition Therapy in Type 2 Diabetes Mellitus and Pre-diabetes: A Series of n-of-1 Superiority Trials	10.3389/fnut.2022.772243	Yes	N-of-1 series (crossover)	Caucasian men with predia-betes/T2DM, age 45-67 y	n=3	DHCR7, PPM1K, MTNR1B, LEPR, SLC30A8, MTHFR SNPs	Genetically-FPG, guided HbA1c MNT vs. conven-tional MNT
S3-10	González-salazar et al. - 2022	Effect of the <i>BCAT2</i> polymorphism (rs11548193) on plasma branched-chain amino acid concentrations after dietary intervention in subjects with obesity and insulin resistance	10.1017/S0007114521002920	Yes	Prospective cohort (1-month diet)	Mexican adults with obesity and IR, BMI ≥30	n=82	BCAT2 rs11548193	Energy-Plasma restricted BCAA, diet (50% AUC carbs) insulin, Matsuda index
S3-11	Deluis et al. - 2021	APOA-5 Genetic Variant rs662799: Role in Lipid Changes and Insulin Resistance after a Mediterranean Diet in Caucasian Obese Subjects	10.1155/2021/1257145	Yes	Intervention (3-month diet)	Caucasian obese adults, BMI ~36, age 25-65 y	n=363	APOA5 rs662799	Hypocaloric Insulin, Mediter-HOMA-IR, ranean diet triglyc-(50% carbs) erides
S3-12	Mojsak et al. - 2021	A Preliminary Study Showing the Impact of Genetic and Dietary Factors on GC–MS-Based Plasma Metabolome of Patients with and without PROX1-Genetic Predisposition to T2DM up to 5 Years Prior to Prediabetes Appearance	10.3390/cimb43020039	Yes	Meal challenge (cross-sectional)	Polish Caucasian nondiabetic men, age ~35 y	n=18	PROX1 rs340874	High-Postprandial carbohydrate metabo-(89% lites; energy) vs. fasting normo-glucose carbohydrate (45%) meals
S3-13	Bauer et al. - 2021	Dietary Macronutrient Intake May Influence the Effects of TCF7L2 rs7901695 Genetic Variants on Glucose Homeostasis and Obesity-Related Parameters: A Cross-Sectional Population-Based Study	10.3390/nu13061936	Yes	Cross-sectional	Caucasian adults, age 18-79 y, ~50% predia-betes/diabetes	n=810	TCF7L2 rs7901695	Macronutrient HbA1c, intake HOMA-IR, (protein, OGTT glu-carbs, fat) cose/insulin via 3-day diaries
S3-14	Zhuang et al. - 2022	Circulating Fatty Acids and Genetic Predisposition to Type 2 Diabetes: Gene-Nutrient Interaction Analysis	10.2337/dc21-2048	Yes	Prospective cohort	Primarily White British	n=95,854	424-SNP GRS (pathway-specific clusters)	Circulating T2D fatty acids incidence (SFA, MUFA, PUFA)
S3-15	Oh et al. - 2025	Identification of Novel Genetic Variants and Food Intake Factors Associated with Type 2 Diabetes in South Korean Adults, Using an Illness–Death Model	10.3390/ijms26062597	Yes	Longitudinal cohort (14 y)	Korean adults, mean age 51 y	Not specified	GCK rs4607517, CAMK2B rs758982, and others	Food intake T2D and (fruits, prediabetes vegetables, incidence meat, grains) via FFQ

Row ID	Study	Title	DOI	Full text retrieved?	Study Type	Population	Sample Size	Genetic Factors	Dietary Factors
S3-16	Munawaroh et al. - 2025	Effectiveness of Personalized Nutrition on Management Diabetes Mellitus Type 2 and Prediabetes in Adults: A Systematic Review	10.2147/DMSO.S528619	Yes	Systematic review	Prediabetes/T2DM adults from multiple countries	8 studies	Not specifically assessed	Personalized HbA1c, nutrition fasting (Mediter-glucose, ranean, PPGR low-fat, MNT)
S3-17	Konstantinidou et al. - 2021	Moving forward the Effects of Gene–Diet Interactions on Human Health	10.3390/nu14183782	Yes	Cross-sectional	Chilean adults, 55% Euro-pean/42% Amerindian ancestry	n=2,828	16-SNP GRSw; TCF7L2 rs7903146, MTNR1B rs10830963	Sugar-Fasting sweetened glucose beverages (SSB) via FFQ
S3-18	Pramono et al. - 2021	The association between vitamin D receptor polymorphisms and tissue-specific insulin resistance in human obesity	10.1038/s41366-021-00744-2	Yes	Intervention (8-wk LCD + 26-wk mainte-nance)	Pan-European obese adults, BMI ~35, age 18-65 y	n=553	VDR rs731236, rs7975232, rs10735810, rs1544410	Diets Tissue-varying in specific IR protein and (HIRI, MISI, glycemic Adipo-IR) index
S3-19	Hosseinpour-niazi et al. - 2022	Effect of TCF7L2 on the relationship between lifestyle factors and glycemic parameters: a systematic review	10.1186/s12937-022-00813-w	Yes	Systematic review	Mixed (non-diabetic, prediabetes, T2DM)	38 studies	TCF7L2 rs7903146 and related variants	Fatty acids, Glucose, fiber, insulin, macronutri-HOMA-IR, ents, HOMA-β Mediter-ranean diet
S3-20	Franzago et al. - 2022	Nutrigenetic variants and response to diet/lifestyle intervention in obese subjects: a pilot study	10.1007/s00592-021-01787-7	Yes	Pilot inter-vention (12-month)	Overweight/obese with T2D or dysg-lycemia, median age 64.5 y	n=18	FTO rs9939609, MC4R rs17782313, LPL rs326, NPY rs16147, IRS-1 rs2943641	Mediterranean Weight, diet (PRED-BMI; IMED HbA1c (not score) detailed)
S3-21	Aronica et al. - 2020	Genetic variants for personalised management of very low carbohydrate ketogenic diets	10.1136/bmjnph-2020-000167	Yes	Narrative review	Mixed pop-ulations (obesity, epilepsy, Alzheimer's)	Multiple studies	LIPF, GYS2, CETP, APOE, and others	Ketogenic HOMA-IR, diets HbA1c (30-50 g (case carbs/day) report)
S3-22	Farrell et al. - 2021	Effect of AMY1 copy number variation and various doses of starch intake on glucose homeostasis: data from a cross-sectional observational study and a crossover meal study	10.1186/s12263-021-00701-8	Yes	Cross-sectional + crossover meal study	Swedish adults, mean age 39 y, mean BMI 25.6	n=1,764 (observa-tional); n=19 (meal study)	AMY1 copy number variation	Habitual Fasting starch glucose; intake; postpran-controlled dial starch glu-doses (40 g, cose/insulin 80 g)
S3-23	Bineid et al. - 2024	A Systematic Review of the Effect of Gene–Lifestyle Interactions on Metabolic-Disease-Related Traits in South Asian Populations	10.1093/nutrit/nuae115	Yes	Systematic review	South Asian popu-lations (India, Pakistan, Sri Lanka, diaspora)	15 studies	FTO, TCF7L2, 10-SNP metabolic GRS	Carbohydrate, FPG, protein, fasting fiber, SFA insulin, intake HbA1c

Row ID	Study	Title	DOI	Full text retrieved?	Study Type	Population	Sample Size	Genetic Factors	Dietary Factors
S3-24	Luukkonen et al. - 2021	The PNPLA3-I148M Variant Confers an Antiatherogenic Lipid Profile in Insulin-resistant Patients	10.1210/clinem/dgaa729	Yes	GWIS (cross-sectional)	European ancestry, UK Biobank, age 40-69 y	n=~340,000	rs147678157, rs3010439, rs62218803, rs140270534, others	30 dietary HbA1c; traits + 8 fasting dietary glucose patterns via FFQ
S3-25	Park - 2021	Interaction of Polygenetic Variants for Gestational Diabetes Mellitus Risk with Breastfeeding and Korean Balanced Diet to Influence Type 2 Diabetes Risk in Later Life in a Large Hospital-Based Cohort	10.3390/jpm11111175	Yes	Cross-sectional (hospital-based cohort)	Korean women aged >40 y	n=34,340 (control) + 384 (GDM)	5-SNP PRS (PTPRD, GPC6, CDKAL1, PRKAG2, PTPRM)	Korean-Fasting style glucose, balanced HbA1c, diet (KBD), T2DM risk Western-style diet via SQFFQ
S3-26	Wuni et al. - 2022	Impact of Lipid Genetic Risk Score and Saturated Fatty Acid Intake on Central Obesity in an Asian Indian Population	10.3390/nu14132713	Yes	Cross-sectional	Asian Indian, mean age 44 y, 42% obese	n=497	CETP rs4783961, LPL rs327/rs3200218 (3-SNP GRS)	Saturated Waist cir-fatty acid cumference intake via (primary); FFQ glycemic outcomes secondary
S3-27	Vimaleswaran - 2021	GeNuIne (gene–nutrient interactions) Collaboration: towards implementing multi-ethnic population-based nutrigenetic studies of vitamin B12 and D deficiencies and metabolic diseases	10.1017/S0029665121002822	Yes	Cross-sectional (multi-ethnic collabora-tion)	Asian Indian, Sri Lankan, Brazilian, Indonesian	Varied (109-900 per cohort)	Metabolic GRS, vitamin D GRS (MTHFR, FTO, TCF7L2, MC4R, others)	Carbohydrate, HbA1c, protein, vitamin D fiber intake status via FFQ
S3-28	Jang et al. - 2025	<i>SLC30A8</i> Rare Variant Modify Contribution of Common Genetic and Lifestyle Factors toward Type 2 Diabetes Mellitus	10.4093/dmj.2024.0830	Yes	Prospective cohort (up to 17 y)	Korean adults, age 40-70 y	n=146,284	SLC30A8 I349F rare variant; T2D PRS	Healthy T2DM Lifestyle incidence Score (diet, sodium, PA, smoking, obesity)
S3-29	Alsulami et al. - 2021	Lower Dietary Intake of Plant Protein Is Associated with Genetic Risk of Diabetes-Related Traits in Urban Asian Indian Adults	10.3390/nu13093064	Yes	Cross-sectional	Urban Asian Indian adults	n=1,062	TCF7L2/FTO 7-SNP and 3-SNP GRS	Plant/animal FPG, protein HbA1c intake via FFQ
S3-30	Alathari et al. - 2022	Interactions between Vitamin D Genetic Risk and Dietary Factors on Metabolic Disease-Related Outcomes in Ghanaian Adults	10.3390/nu14132763	Yes	Cross-sectional	Ghanaian adults, age 25-60 y, mean BMI 26.6	n=302	8-SNP vitamin D GRS (VDR, DHCR7, CYP2R1, others)	Fiber, fat, HbA1c, SFA intake BMI via 24-h recall
S3-31	Alanazi et al. - 2024	A Systematic Review of the Gene–Lifestyle Interactions on Metabolic Disease-Related Outcomes in Arab Populations	10.3390/nu16152519	Yes	Systematic review	Arab popu-lations (Lebanon, Algeria, UAE)	5 studies	FTO rs9939609, TCF7L2 rs7903146, MC4R rs17782313, MTHFR rs1801133	Energy, FPG, T2D SFA, risk, BMI, Mediter-WC ranean diet adherence, desserts via FFQ
S3-32	Sekar et al. - 2025	High Polyunsaturated Fatty Acid Intake Attenuates the Genetic Risk of Higher Waist Circumference in a Sri Lankan Adult Population	10.3390/nu17172866	Yes	Cross-sectional	Sri Lankan adults, age 25-50 y	n=105	10-SNP metabolic GRS (TCF7L2, CAPN10, FTO, KCNJ11, MC4R)	PUFA Waist cir-intake via cumference FFQ (primary)

Row ID	Study	Title	DOI	Full text retrieved?	Study Type	Population	Sample Size	Genetic Factors	Dietary Factors
S3-33	Rao et al. - 2025	Interaction between coffee consumption and polygenic risk score in relation to diabetes: insights from the Maastricht study	10.1007/s00394-025-03782-y	Yes	Cross-sectional	Southern Nether-lands, median age 61 y	n=7,668	423-SNP PRS for T2D	Coffee con- Prediabetes sumption and T2DM via FFQ prevalence
S3-34	Parnell et al. - 2025	CC Genotype at TCF7L2 Diabetes Risk Locus rs7903146 Directs a Coordinated Fatty Acid Response to a Mediterranean Diet Intervention: A Randomized Controlled Trial	10.1159/000542468	Yes	RCT crossover (1-wk per diet)	Overweight/obese adults, BMI 26-34, age 18-70 y	n=35	TCF7L2 rs7903146	Mediterranean Fatty acid vs. low-fat metabolites diet (lipid focus)
S3-35	Song et al. - 2022	Gene–Environment Interaction on Type 2 Diabetes Risk among Chinese Adults Born in Early 1960s	10.3390/genes13040645	Yes	Cross-sectional	Chinese adults born in early 1960s, age ~50 y	n=2,216	Multiple SNPs (rs340874, rs5015480, rs7612463, others)	Meat/poultry, FPG, cere-fasting als/beans insulin, intake via T2D/IFG/IGT 24-h recall
S3-36	Maciejewska-skrendo et al. - 2022	The Influence of the Differentiation of Genes Encoding Peroxisome Proliferator-Activated Receptors and Their Coactivators on Nutrient and Energy Metabolism	10.3390/nu14245378	Yes	Systematic review	Not specified	Multiple studies	PPARG rs1801282, PPARD rs2016520, PPARGC1A rs8192678	High-fat, Fasting low-energy, insulin, PUFA-rich insulin diets sensitivity
S3-37	Hardy et al. - 2023	Analysis of ancestry-specific polygenic risk score and diet composition in type 2 diabetes	10.1371/journal.pone.0285827	Yes	Cross-sectional	European Americans (83.3%) and African Americans (16.7%)	n=9,393	Ancestry-specific PRS (~121K- 265K variants)	Carbohydrate T2DM and protein status intake (% calories) via FFQ
S3-38	Billings et al. - 2024	Increased Genetic Risk for β-Cell Failure Is Associated With β-Cell Function Decline in People With Prediabetes	10.2337/db23-0761	Yes	RCT (Diabetes Prevention Program)	Overweight/obese prediabetic adults, mean BMI ~34, diverse ethnicity	n=2,647	Partitioned PPS (β-cell, proinsulin, liver/lipid, obesity, lipodystro-phy; 30 SNPs for β-cell)	Intensive Insulinogenic lifestyle vs. index, CIR, metformin T2D vs. placebo incidence
S3-39	Hur et al. - 2022	Association of Polygenic Variants with Type 2 Diabetes Risk and Their Interaction with Lifestyles in Asians	10.3390/nu14153222	Yes	Cross-sectional + replication	Korean adults aged >40 y	n=58,701	8-SNP PRS (CDKAL1, KCNQ1, GLIS3, others)	Energy Fasting intake glucose, (EER), T2DM risk Western-style diet, KBD via SQFFQ
S3-40	Adamska-patruno et al. - 2021	An Association between Diet and MC4R Genetic Polymorphism, in Relation to Obesity and Metabolic Parameters—A Cross Sectional Population-Based Study	10.3390/ijms222112044	Yes	Cross-sectional	Caucasian Polish adults, age 18-79 y, ~50% predia-betes/diabetes	n=819	MC4R rs17782313, rs12970134, rs633265, rs1350341	Protein, Fasting carbohy-glucose, drate, fat OGTT, intake via HOMA-IR, 3-day HbA1c diaries

Row ID	Study	Title	DOI	Full text retrieved?	Study Type	Population	Sample Size	Genetic Factors	Dietary Factors
S3-41	Al-odinan et al. - 2025	Interaction between the TCF7L2 gene and dietary intake on metabolic syndrome risk factors among Saudi Arabian adults	10.3389/fnut.2025.1513088	Yes	Cross-sectional	Saudi adults, age 20-55 y, BMI 18-35	n=271	TCF7L2 rs7903146	Total Fasting energy, car-insulin bohydrate, SFA, MUFA, PUFA via FFQ
S3-42	Sekar et al. - 2024	A Novel Interaction between a 23-SNP Genetic Risk Score and Monounsaturated Fatty Acid Intake on HbA1c Levels in Southeast Asian Women	10.3390/nu16173022	Yes	Cross-sectional	Minangkabau women, age 25-60 y	n=106	23-SNP GRS (19 genes including TCF7L2, FTO, MC4R, PPARG)	MUFA, HbA1c protein, fiber, fat intake via SQ-FFQ
S3-43	Madhu et al. - 2022	TCF7L2 gene associated postprandial triglyceride dysmetabolism- a novel mechanism for diabetes risk among Asian Indians	10.3389/fendo.2022.973718	Yes	Cross-sectional (fat challenge test)	Asian Indian adults, age 20-60 y, mean BMI ~28	n=620	TCF7L2 rs7903146	Standardized Postprandial fat triglyc-challenge erides, meal glucose, HOMA-IR
S3-44	Lam et al. - 2025	Integrative Roles of Functional Foods, Microbiotics, Nutrigenetics, and Nutrigenomics in Managing Type 2 Diabetes and Obesity	10.3390/nu17040608	Yes	Systematic literature review	Mixed (T2DM, obesity)	129 core articles	FTO, PPARγ, ADIPOQ, TCF7L2	Functional Insulin foods, sensitivity, Mediter-metabolic ranean diet, outcomes dietary fat
S3-45	Molani-Gol et al. - 2025	The interaction of vitamin D supplementation with Omentin-1 gene polymorphism on metabolic factors and anthropometric indices in women with prediabetes: a study protocol for a double-blind randomized controlled trial	10.1186/s12906-025-05034-2	Yes	Double-blind RCT	Iranian women with prediabetes, age 18-65 y, 70.8% over-weight/obese	Not specified	Omentin-1 Val109Asp (rs2274907)	Vitamin D FPG, supplemen-insulin, tation; HOMA-IR, dietary HbA1c, intake via HOMA-β 24-h recall
S3-46	Kapellou et al. - 2025	Gene–Diet Interactions in Diabetes Mellitus: Current Insights and the Potential of Personalized Nutrition	10.3390/genes16050578	Yes	Narrative review	Diverse (multiple ethnicities)	Multiple studies	LRRC24, CCDC40, LIN9, rs79762542; PRS (67 and 424 SNPs)	Mediterranean FPG, diet, low-HbA1c, inflammatory T2DM diet, carbo-incidence hydrate substitu-tion
S3-47	Li et al. - 2022	Association of alcohol drinking with incident type 2 diabetes and pre-diabetes: The Guangzhou Biobank Cohort Study	10.1002/dmrr.3548	Yes	Prospective cohort (~4 y)	Chinese adults aged >50 y	n=15,716	ALDH2 rs671, ADH1B rs1229984	Alcohol consumption (not T2D/IFG diet-specific)
